# The Expression and Function of lincRNA-154324 and the Adjoining Protein-Coding Gene *vmp1* in the Caudal Fin Regeneration of Zebrafish

**DOI:** 10.3390/ijms23168944

**Published:** 2022-08-11

**Authors:** Jing Li, Wenjun Wen, Shuqiang Zhang, Chune Zhou, Yiyi Feng, Xiaoyu Li

**Affiliations:** 1 The School of Medical Humanities, Xinxiang Medical University, Xinxiang 453003, China; 2 Henan International Joint Laboratory of Aquatic Toxicology and Health Protection, College of Life Science, Henan Normal University, Xinxiang 453007, China

**Keywords:** zebrafish, caudal fin, regeneration, lncRNA, *vmp1*

## Abstract

Caudal fin regeneration is regulated by a variety of mechanisms, but the role of long non-coding RNA (lncRNA) has rarely been studied. The present study aimed to describe the landscape of lncRNAs during caudal fin regeneration using whole transcriptome sequencing, and then to conduct a functional study on the target lncRNAs using real-time fluorescent quantitative PCR (RT-qPCR), in situ hybridization, and the CRISPR/Cas9 method for lncRNA gene knockout. The results of the transcriptome sequencing showed that a total of 381 lncRNAs were differentially expressed, among which ENSDART00000154324 (lincRNA-154324) was found to be highly related to caudal fin regeneration, and thus it was chosen as the target lncRNA for the subsequent functional study. The results regarding the temporal and spatial expression of lincRNA-154324 and the gene knockout results from CRISPR/Cas9 indicated that lincRNA-154324 is involved in the caudal fin regeneration of zebrafish. Importantly, we serendipitously discovered that the cis correlation coefficient between lincRNA-154324 and its neighboring gene vacuole membrane protein 1 (*vmp1*) is extremely high, and they are essential for the process of caudal fin regeneration. Moreover, studies have found that *vmp1* plays an important role in protein secretion, organelle formation, multicellular development, and autophagy. Collectively, our result may provide a framework for the identification and analysis of lncRNAs involved in the regeneration of the zebrafish caudal fin.

## 1. Introduction

Mammals, including humans, cannot regenerate damaged limbs, but bony fish (such as the model organism zebrafish) possess strong damage-response capabilities, in which all types of fin tissues can be regenerated after loss. This is an interesting and noteworthy function, since the loss of normal motor function in the limbs caused by hereditary or acquired limb diseases has a huge impact on people’s quality of life and causes a huge economic burden. In the past few decades, much work has been devoted to exploring the key regulators of caudal fin regeneration and developing treatments for limb loss. Although these studies have shown that there are many regeneration regulatory factors and mechanisms that show strong potential, the specific mechanism of action in regeneration is still an unsolved mystery [1]. In addition, the zebrafish genome shares more than 70% homology with the human genome [2]. Therefore, exploring the regeneration mechanism of species with strong regeneration abilities has important scientific significance for solving this mystery, and it is also a hot and difficult point in the fields of life science and medical research.

As a model organism, zebrafish have strong regeneration abilities, especially for the caudal fin [3,4]. Zebrafish caudal fin is easy to manipulate and has well-established forward and reverse genetics, which makes it a popular model system for studying regeneration. The zebrafish caudal fin is formed through the construction of the exoskeleton and endoskeleton [5]. The endoskeleton is developed from cartilage tissue and cannot be regenerated after being removed, while the exoskeleton is developed from dermal tissue and retains the ability to regenerate. The exoskeleton fins consist of two types of fin rays;, namely, lepidotrichia and actinotrichia. The lepidotrichia are connected by multiple bone-like joints and each joint is formed of two symmetrical halves facing each other. The two halves are hollow and contain blood vessels, nerves, and connective tissues. Actinotrichia originate from the end of the lepidotrichia and are rod-shaped with highly polymerized collagen. Fin growth in zebrafish is promoted by increasing the number of joints, which occurs throughout their lives [6,7]. The process of caudal fin regeneration is extremely complex, and it is regulated by cross-talk between a variety of genes [8,9,10,11,12]. More importantly, the bony fin of zebrafish is very similar to a human limb in early development [13]. Therefore, fin regeneration can be used as a good model to investigate the molecular mechanism of human limb regeneration.

Vacuole membrane protein 1 (*vmp1*) is a macromolecular transmembrane protein. *Vmp1* transcript is about 2135 bp in length and is located on zebrafish chromosome 15 (chromosome 15: 17,343,319–17,373,352), with 12 exons. Studies have indicated that *vmp1* plays an important role in protein secretion, organelle formation, multicellular development, and autophagy formation [14]. Therefore, we wondered whether *vmp1* also participates in the process of zebrafish caudal fin regeneration. However, there are few reports about its role in caudal fin regeneration. Recent studies on colorectal cancer showed that miR-21 accumulated in cancer cells depleted of *vmp1*, indicating that *vmp1* can regulate cancer cell proliferation, invasion, and metastasis through miR-21 [15]. Therefore, we speculated that *vmp1* may be regulated by its downstream non-coding RNA in caudal fin regeneration.

In mammals, most of the genome can be transcribed into RNA. Except for a few of these transcribed RNAs that can encode proteins, more than 98% are non-coding RNAs (ncRNAs) that do not have the potential to encode proteins [16,17,18]. With the continuous improvement of high-throughput sequencing technology and genome projects, more and more ncRNAs have been discovered and identified. With the deepening of the understanding of ncRNAs, researchers have gradually realized that ncRNAs are not a “noise sequence” as we thought before, but that some elements have important regulatory effects on gene networks. ncRNAs can be divided into long non-coding RNAs (lncRNAs) and microRNAs (miRNAs) according to the length of their transcripts [19]. LncRNAs are a type of ncRNA with lengths greater than 200 nt that play an important role in transcriptional silencing, transcriptional activation, chromosome modification, and nuclear transport. Association analyses of lncRNA and protein coding genes (mRNA) include antisense analysis, cis action analysis, and trans action analysis. In these analyses, the principle of cis relationships used for target gene prediction is that the function of an lncRNA is related to its neighboring mRNA. The lncRNA located upstream of the protein-coding gene may overlap with the promoter or other cis-acting elements of the co-expressed gene, thereby regulating the expression of adjacent genes at the transcriptional or post-transcriptional level [20]. Studies have indicated that ncRNAs have an extremely important regulatory role in human diseases, animal growth, and regeneration [21,22,23,24,25,26]. Recently, several studies have shown that miRNA-133 and miRNA-203 can affect caudal fin regeneration in zebrafish by regulating the target genes (mps1 and lef1) [27,28]. However, few studies are available regarding the expression and function of lncRNAs in the caudal fin regeneration of fish. The present study aimed to investigate the expression and function of lncRNAs (ENSDART00000154324, lincRNA-154324) and their neighboring gene (*vmp1*) in caudal fin regeneration in adult zebrafish in order to partly reveal the roles of lincRNA-154324 and *vmp1* in regeneration.

## 2. Results

### 2.1. Identification of lncRNAs Related to Caudal Fin Regeneration by RNA-Seq

**Figure 1:**
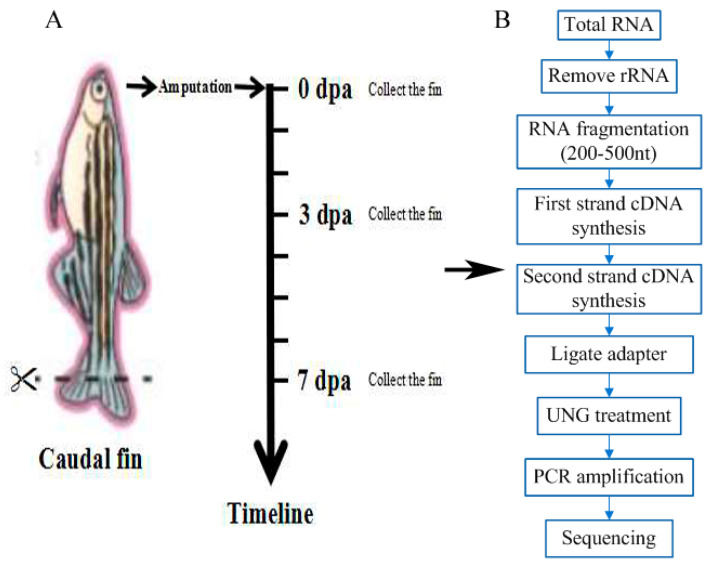
Schematic diagram of the experimental process. (**A**) Schematic diagram of the time points of the zebrafish caudal fin sampling. (**B**) Schematic diagram of the experimental process. CK0dpa, control group at 0 days post-amputation; T3dpa and T7dpa, caudal fin regeneration at 3 and 7 days post-amputation, respectively.

In order to identify the expression of lncRNAs during caudal fin regeneration in zebrafish, a cDNA library was constructed for the caudal fin tissue regenerated at 0, 3, and 7 day post-amputation (dpa), and RNA-seq was subsequently performed (Figure 1A,B). After the sequencing data were filtered, the percentage of data reaching the quality level of Q20 (sequencing accuracy rate of 99%) ranged from 97% to 98%, and the percentage of data reaching the quality level of Q30 (sequencing accuracy rate of 99.9%) ranged from 93% to 94%. The percentage of GC content varied from 51% to 53%. The quality of the constructed transcriptome library data was good, as shown in Table 1, which ensured the accuracy of the next experiment.

### 2.2. LncRNA Expression Pattern during the Process of Caudal Fin Regeneration

**Figure 2:**
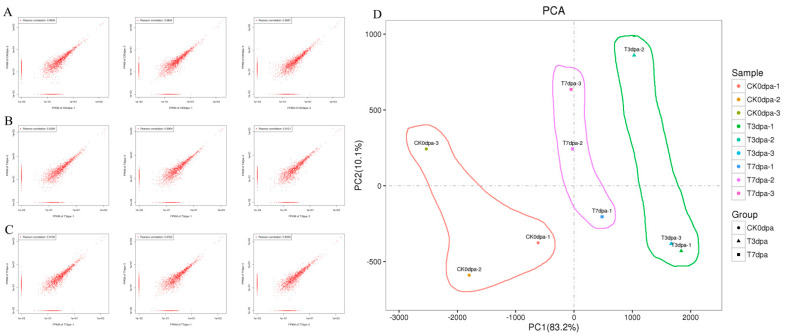
Quality analysis on the transcriptome sequencing. (**A**–**C**) The correlation coefficients of the paired repeatability test. The closer the correlation between two parallel experiments of the same sample was to 1, the higher the repeatability. CK0dpa, control group at 0 days post-amputation; T3dpa and T7dpa, caudal fin regeneration at 3 and 7 days post-amputation, respectively. (**D**) The principal component analysis (PCA) for each sample. CK0dpa, control group at 0 days post-amputation; T3dpa and T7dpa, caudal fin regeneration at 3 and 7 days post-amputation, respectively.

In order to investigate the expression characteristics of ncRNAs in the process of caudal fin regeneration, the expression of lncRNAs was recorded in fragments per kilobase of transcript per million mapped reads (FPKM). The correlation analysis of the results of two parallel experiments made it possible to obtain an evaluation of the reliability of the experimental results and the stability of operation. As shown in the Figure 2A–C, the closer the correlation between two parallel experiments for the same sample was to 1, the higher the repeatability. In addition, the principal component analysis (PCA) showed that the lncRNA profiles in regenerated caudal fins at the same time point were close to each other (Figure 2D), which once again emphasized the consistency of the biological samples at the same time point. Interestingly, in the results from the PCA analysis, the lncRNA profile was different at each time point, indicating a significant regulation of gene expression during the caudal fin regeneration. The caudal fin of the 3 dpa group showed the greatest difference in lncRNA expression compared to the 0 dpa control group, and there was a process whereby expression returned to the baseline (0 dpa) expression profile in the late regeneration period at 7 dpa (Figure 2D).

**Figure 3:**
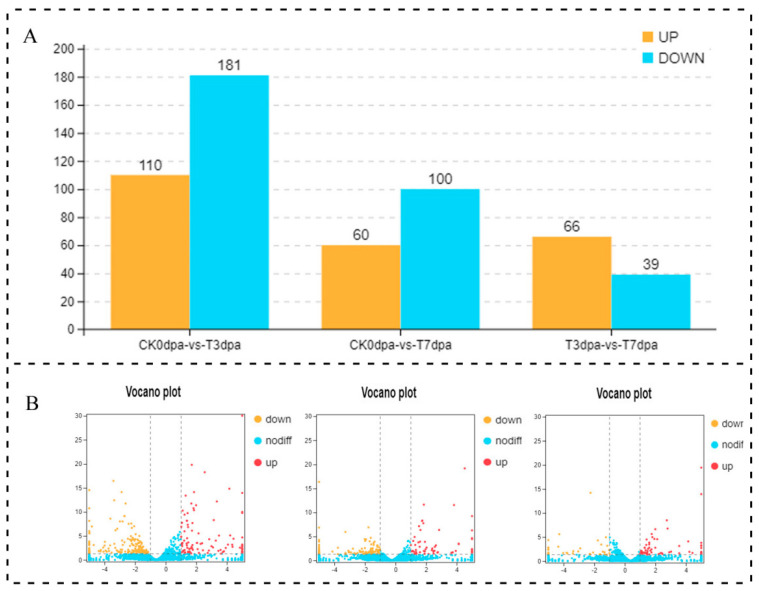
The statistical analysis on the DE lncRNAs at the different stages of caudal fin regeneration. (**A**) The numbers of up-regulated and down-regulated lncRNAs at different regeneration stages are showed in a bar graph. (**B**) The numbers of up-regulated and down-regulated lncRNAs at different regeneration stages are showed in volcano plots. The abscissa represents the logarithmic value of the difference multiple of two samples (CK0dpa vs. T3dpa, CK0dpa vs. T7dpa, T3dpa vs. T7dpa), and the ordinate represents the negative log10 value of the FDR of the two samples. The red (sample−2 expression is up-regulated relative to sample−1) and yellow (the expression level is down-regulated) colors indicate that there is a difference in gene expression (judgment criterion was FDR < 0.05, and the difference multiple was more than 2), and the black color indicates no difference. CK0dpa, control group at 0 days post-amputation; T3dpa and T7dpa, caudal fin regeneration at 3 and 7 days post-amputation, respectively.

Through the whole transcriptome resequencing, 3966 lncRNA transcripts were obtained in total. edgeR software was used to analyze the DE of lncRNAs between group, and the screening conditions were |log2FC| > 2 and *p* < 0.05. The amount of DE lncRNAs differed between different groups (Figure 3, Tables S1–S3). For example, compared with 0 dpa (control group), 110 lncRNAs were up-regulated at 3 dpa, while 181 lncRNAs were down-regulated at this time point (Figure 3A,B). However, there were only 60 up-regulated lncRNAs and 100 down-regulated lncRNAs at 7 dpa (Figure 3A,B). Compared to 3 dpa, the number of DE lncRNAs was the smallest at 7 dpa (Figure 3A,B). This result suggests that lncRNAs might undergo complex changes in the amputated caudal fin, especially at 3 dpa.

**Figure 4:**
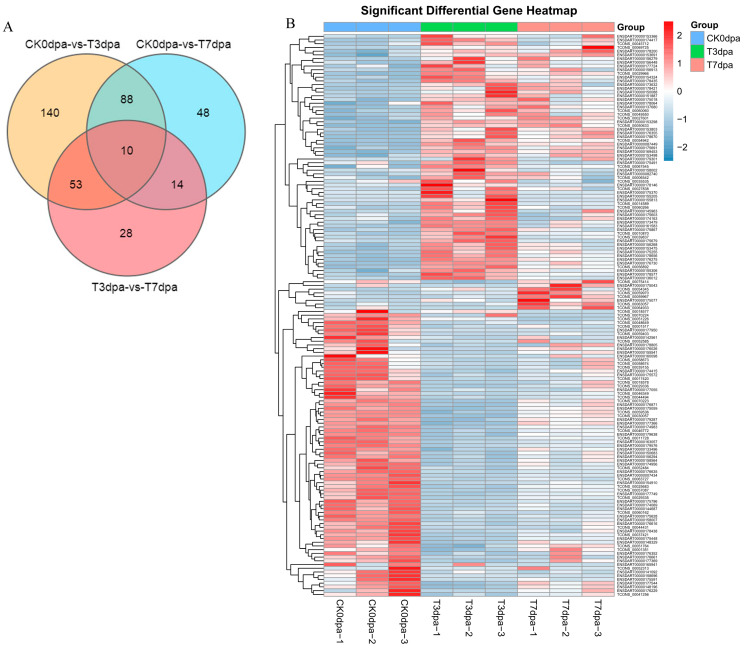
The statistical analysis on the DE lncRNAs at different stages of caudal fin regeneration. (**A**) Venn diagram showing the DE lncRNAs at three stages of caudal fin regeneration. (**B**) Heat map showing the DE lncRNA expression levels during caudal fin regeneration at 0 dpa, 3 dpa, and 7 dpa. CK0dpa, control group at 0 days post-amputation; T3dpa and T7dpa, caudal fin regeneration at 3 and 7 days post-amputation respectively.

In order to explore similarities in lncRNA expression, a clustering heat map analysis of gene expression patterns was used to investigate the differential expression of lncRNAs (Figure 4B). In order to further study the interaction of DE lncRNAs at different regeneration stages, a Venn diagram was constructed for the DE lncRNAs (Figure 4A). There were a total of 10 DE lncRNAs at these three regeneration stages.

### 2.3. The GO and KEGG Enrichment Analyses

**Figure 5:**
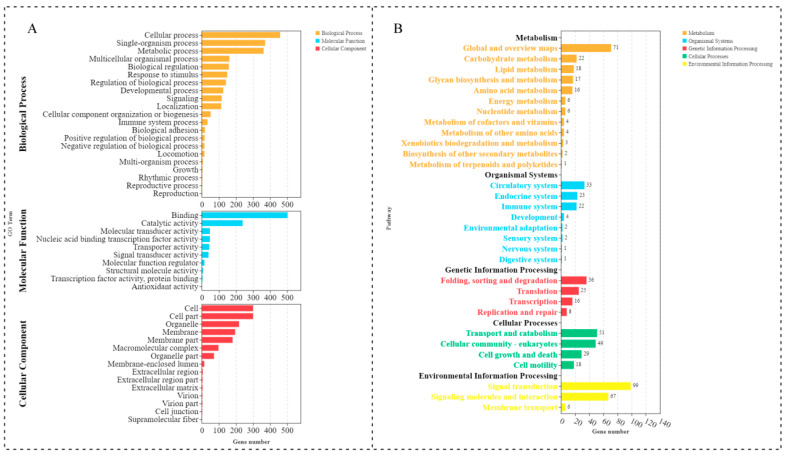
GO and KEGG enrichment analyses for the DE lncRNA cis-targeted genes. (**A**) Histogram of significant GO enrichment in DE lncRNAs. Yellow represents biological processes, red represents cellular components, and blue represents molecular functions. (**B**) KEGG pathway enrichment in DE lncRNAs.

In order to further understand the specific roles of these DE lncRNAs in the process of caudal fin regeneration, GO and KEGG pathway enrichment analyses were performed for the target genes of the DE lncRNAs (the cis target genes). The results of the GO enrichment analysis of the DE lncRNA target genes showed that, in the 20 biological processes where DEG target genes were significantly enriched, most genes were significantly enriched in the GO terms related to caudal fin regeneration, including biological regulation (GO:0065007), cell process (GO:0009987), development process (GO:0032502), and metabolic process (GO:0008152) (Figure 5A, Table S4). The KEGG pathway analysis results showed that DE lncRNA target genes were significantly enriched in the Wnt signaling pathway, cell adhesion signaling pathway, proteasome signaling pathway, MAPK signaling pathway, and other classic signaling pathways related to development and regeneration (Figure 5B, Table S5). We analyzed the correlation between differentially expressed lncRNA and differentially expressed mRNA. Interestingly, lncRNA-154324 had a cis correlation with vmp1, and its cis correlation coefficient was above 0.8 (the cis correlation coefficient was calculated according to the Pearson correlation coefficient of the lncRNA and mRNA based on the expression levels of the lncRNA and mRNA) (Table S6).

### 2.4. The Expression of the Regeneration-Related lncRNAs

**Figure 6:**
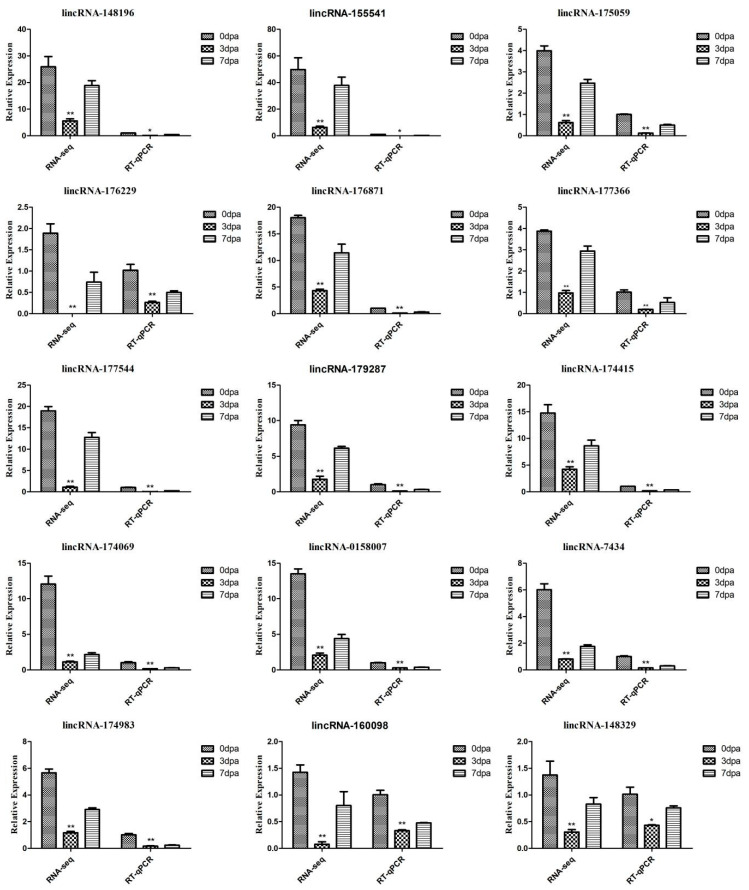
Verification of the differentially expressed lncRNAs with RT-qPCR: the relative expression of lncRNAs in caudal fin regeneration. Data (mean ± SEM) are representative results derived from three independent experiments. (**) *p* < 0.01, (*) *p* < 0.05, when comparing to 0 dpa.

In accordance with the significance of the differential expression of lncRNA and the results of the bioinformatics analysis, we selected the interesting lncRNAs (FDR < 0.05, |log2FC| > 1, DEG shared between groups and bioinformatics analysis showing association with regeneration) to study their expression and to explore the expression characteristics in key processes of zebrafish caudal fin regeneration. Then, RT-qPCR was conducted to detect and verify the expression of related DE lncRNAs during the critical periods of caudal fin regeneration. A similar expression pattern was found to that from the RNA-seq (Figure 6).

### 2.5. The Temporal and Spatial Expression of lincRNA-154324 and the Cis Neighboring Gene vmp1 during Caudal Fin Regeneration

**Figure 7:**
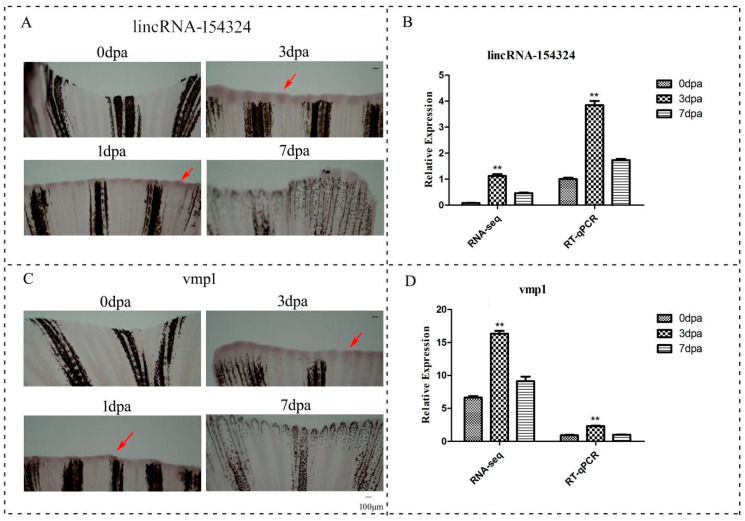
The temporal and spatial expression of lincRNA-154324 and *vmp1* in caudal fin regeneration. (**A**) 0 dpa–7 dpa: lincRNA-154324 spatial expression in zebrafish caudal fin for different processes of regeneration, as shown using WISH. Red arrow: pointing to the positive site. Scale bars: 100 μm. (**B**) The relative expression of lincRNA-154324 in caudal fin regeneration, as shown using RT-qPCR and RNA-seq. Data (mean ± SEM) are representative results derived from three independent experiments. (**) *p*< 0.01, when comparing to 0 dpa. (**C**) 0 dpa–7 dpa: *vmp1* spatial expression in zebrafish caudal fin regeneration, as shown using WISH. Red arrow: pointing to the positive site. Scale bars: 100 μm. (**D**) The relative expression of *vmp1* in caudal fin regeneration, as shown using RT-qPCR and RNA-seq. Data (mean ± SEM) are representative results derived from three independent experiments. (**) *p* < 0.01, when comparing to 0 dpa.

The whole-mount in situ hybridization (WISH) results showed that lincRNA-154324 was mainly expressed in the wound epidermis at 1 dpa, and the expression gradually increased and reached the highest level at 3 dpa (Figure 7A,B). Subsequently, the expression level began to decline (Figure 7A 7 dpa). The expression trend for *vmp1*, the cis neighboring gene of lincRNA-154324, was similar to that of the lncRNA (Figure 7C,D). It is particularly worth noting that the expression of these genes was initially expressed in the wound epidermis and then gradually transferred to the blastema tissue (Figure 7A,C), indicating that they might play a role in the initiation and proliferation of fin regeneration.

### 2.6. LincRNA-154324 Is Involved in the Development and Growth of Caudal Fin

**Figure 8:**
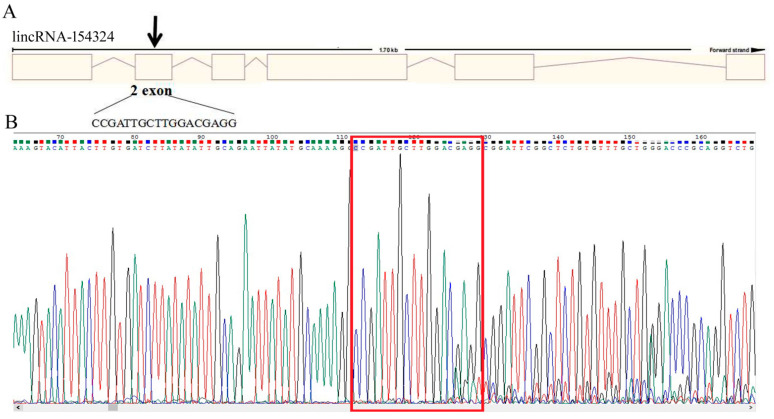
The sequence detection results for lincRNA-154324 knockout. (**A**) Gene architecture of lincRNA-154324 showing the target site of the SgRNA. (**B**) The sequence detection results for lincRNA-154324 knockout for embryos at 24 hpf. The red box indicates the knockout target site area.

**Figure 9:**
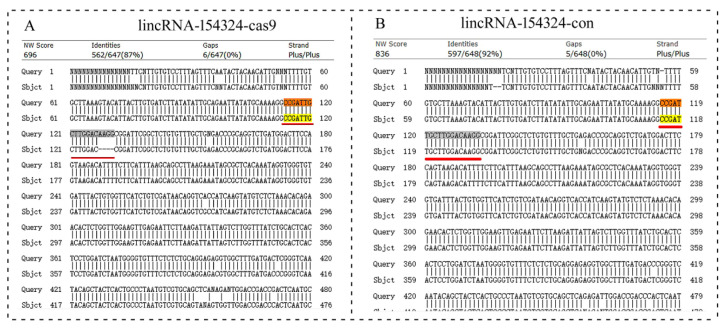
The sequence detection results for lincRNA-154324 knockout. (**A**) Alignment of caudal fin sequences from the control and the lincRNA-154324-cas9-knockout F0-generation adult zebrafish. (**B**) Alignment of caudal fin sequences from the control and the lincRNA-154324-con F0-generation adult zebrafish. Highlight, red line and red box: indicates the knockout target site area.

In order to explore the biological function of lincRNA-154324 in caudal fin regeneration in zebrafish, a CRISPR/Cas9 knockout analysis was performed with the fish zygotes at the one-cell stage. The knockout target site of lincRNA-154324 was set to the fourth exon (Figure 8A). To test the efficiency and effectiveness of knockout, the injected embryos were collected at 24 h post-fertilization (hpf) following injection of Cas9-SgRNA-RNP (con, con-cas9, and cas9: 10 each) for rapid DNA extraction and sent to a company (GENEWIZ, Tianjin, China) for sequencing. Sequencing results showed that there was regular peak nesting at the target of the lincRNA-154324-cas9 group, which suggests that the knockout might have been successful (Figure 8B). At 2 to 3 months after the gene knockout, the caudal fins of the control group and the knockout group were clipped and sequenced. The sequence comparison showed that there were four base deletions in the sequence at the target site of the knockout group (Figure 9A). Therefore, the F0-generation, which successfully obtained the knockout mutation with the deletion of four bases, had to be reared separately to prepare for further subsequent breeding of homozygotes.

**Figure 10:**
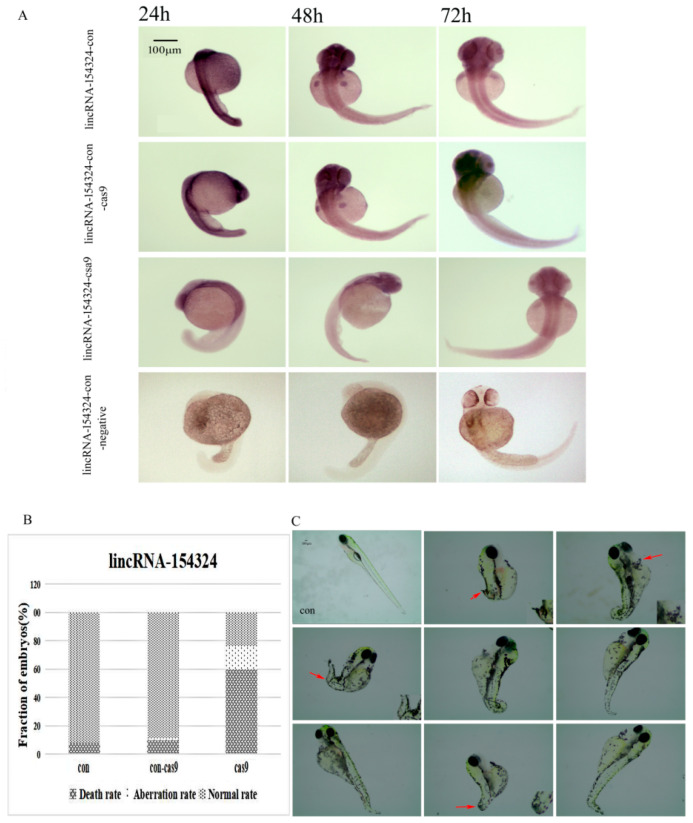
The expression and morphology of the lincRNA-154324 knockout larvae. (**A**) LincRNA-154324 spatial expression in the lincRNA-154324-con, lincRNA-154324-con-cas9 (a control group in which only cas9 was injected), lincRNA-154324-cas9, and lincRNA-154324-con-negative (a negative control group for WISH) groups at different stages of zebrafish embryogenesis. Scale bars: 100 μm. (**B**) Frequency of CRISPR/Cas9-micro-injection phenotypes in injected embryos. (**C**) The morphological results after lincRNA-154324 gene knockout at 7 dpf. con: control group. Red arrow: pointing to the deformed part. Scale bars: 100 μm.

In order to further verify the expression of lincRNA-154324 after knockout, we conducted a WISH experiment and the results showed that the expression of lincRNA-154324 in the lincRNA-154324-cas9 group was reduced in some parts compared to the control group (con and con-cas9) (Figure 10A). Specifically, the expression level in the spine during the development of the caudal fin was low, and it was found that the embryonic development of the knockout group was slower than that of the control group when the material was taken at the same time point (Figure 10A). At 7 days post-fertilization (dpf) with zebrafish embryo CRISPR/Cas9 knockout injection, the embryos in the zebrafish knockout group developed slowly, and a shortened body length, curled-up caudal fin, and larval fish without caudal fins were observed. Moreover, we also found that the fish pericardium was enlarged and congested, and the eyes were bulged (Figure 10C). In addition, the effects of the lncRNA knockout on the death and deformity rates of zebrafish embryos were calculated, and the results showed that the lincRNA-154324 knockout group (cas9) had a death rate of 60% and a malformation rate of 16%, while the control group had low rates of death and malformation (Figure 10B).

### 2.7. Vmp1 Plays a Role in the Development and Growth of the Caudal Fin

**Figure 11:**
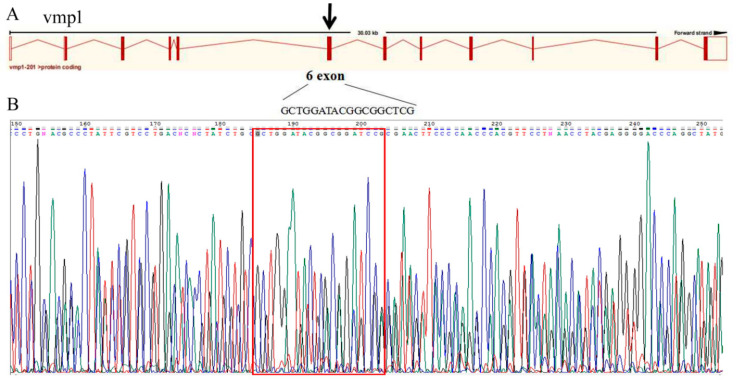
The sequence detection results for *vmp1* knockout. (**A**) Gene architecture of *vmp1*, showing the target site of the SgRNA. (**B**) The sequence detection results for *vmp1* knockout for 24 hpf embryos. The red box indicates the knockout target site area.

**Figure 12:**
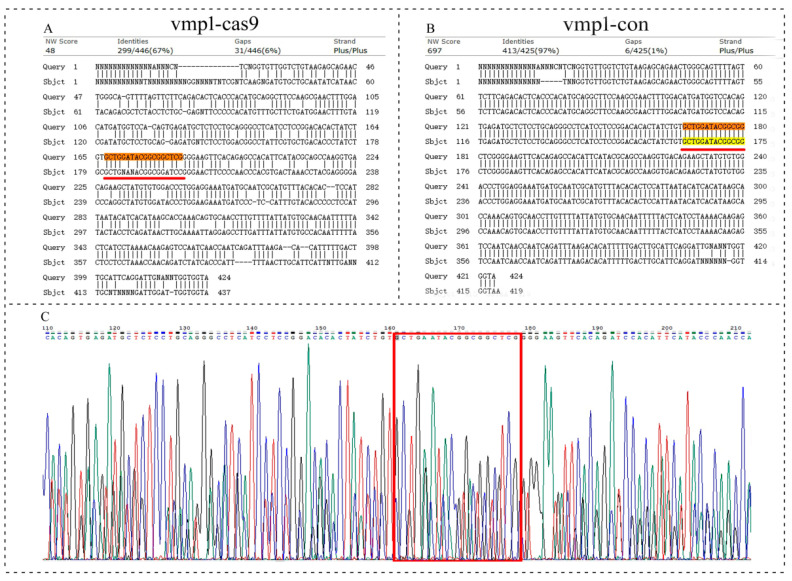
The sequence detection results for *vmp1* knockout. (**A**) Alignment of caudal fin sequences from the control and the *vmp1*-cas9-knockout F0-generation adult zebrafish. (**B**) Alignment of caudal fin sequences from the control and the *vmp1*-con F0-generation adult zebrafish. (**C**) Sequencing of the 24 hpf embryos from the *vmp1*-cas9-knockout F1-generation fish. Highlight red line and red box: indicates the knockout target site area.

The basic principle of cis-target-gene prediction for lncRNA is that the function of an lncRNA is related to its neighboring protein-coding genes. Therefore, we applied CRISPR-Cas9 technology to study the function of the lincRNA-154324 cis target gene *vmp1*. The knockout target site of *vmp1* was set to the sixth exon (Figure 11A). The results of the gene knockout showed that there was regular peak nesting at the target points in the *vmp1*-cas9 group, suggesting that the knockout might have been successful (Figure 11B). At 2 to 3 months after the gene knockout, the caudal fins of the control group and the knockout group were clipped and sequenced. After comparing the sequences, it was found that the knockout group had four base mismatches at the target site, and there were multiple base mismatches and deletions behind the target site (Figure 12A). In addition, the adult F0 generation that was knockout-positive for this gene was crossed with wild-type fish, and the F1-generation with peak nesting at the knockout target was obtained (Figure 12C).

**Figure 13:**
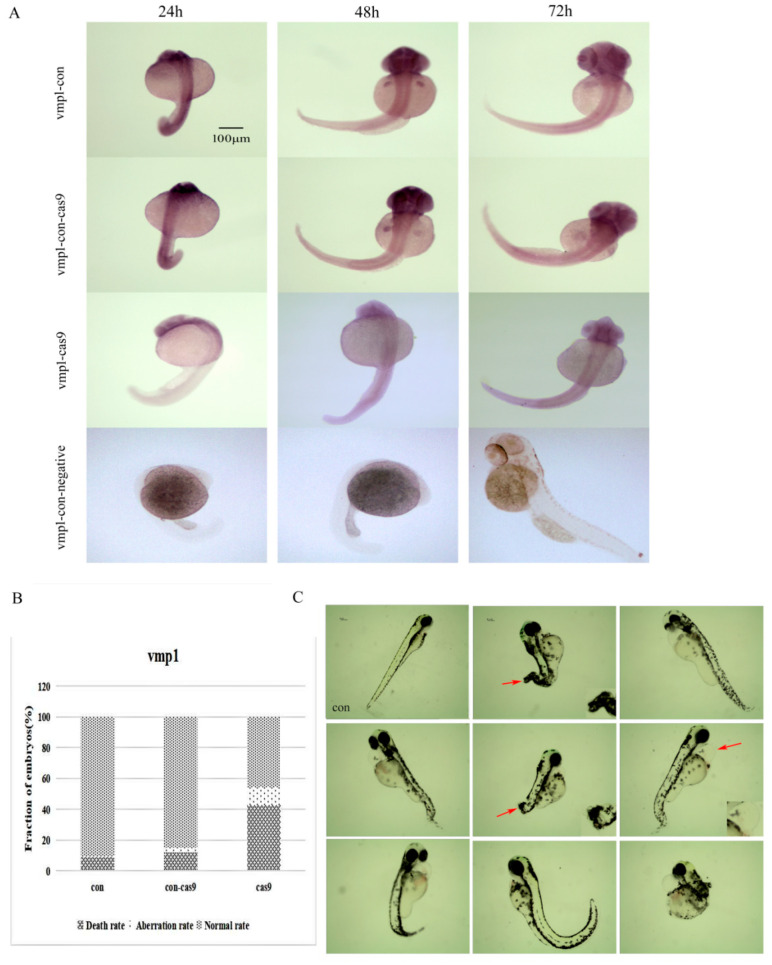
The expression and morphology of the *vmp1* knockout larvae. (**A**) *Vmp1* spatial expression in the *vmp1*-con, *vmp1*-con-cas9 (a control group in which only cas9 was injected), *vmp1*-cas9, and *vmp1*-con-negative (a negative control group for WISH) groups at different stages of zebrafish embryogenesis. Scale bars: 100 μm. (**B**) Frequency of CRISPR/Cas9-micro-injection phenotypes in injected embryos. (**C**) The morphological results after *vmp1* gene knockout at 7 dpf. con: control group. dpf: days post-fertilization. Red arrow: pointing to the deformed part. Scale bars: 100 μm.

In order to further verify the expression of *vmp1* after knockout, WISH was performed and the results showed that the expression of *vmp1* in the knockout group was significantly reduced, especially in the development of the caudal fin, compared with the control groups (con and con-cas9). Moreover, spine malformation was observed in the *vmp1*-cas9 group (Figure 13A). We also found developmental lag, shortened body length, and curled-up tails in the fish embryos from the knockout group at 7 dpf following the *vmp1* gene knockout injection (Figure 13C). The results of the statistical analysis indicated 43% mortality and 11% deformity rates in the *vmp1*-cas9 group, but there were only a few deaths and deformities in the control group (Figure 13B).

### 2.8. Knocking out lincRNA-154324 or vmp1 Altered the Expression of the Marker Genes of Caudal Fin Regeneration

**Figure 14:**
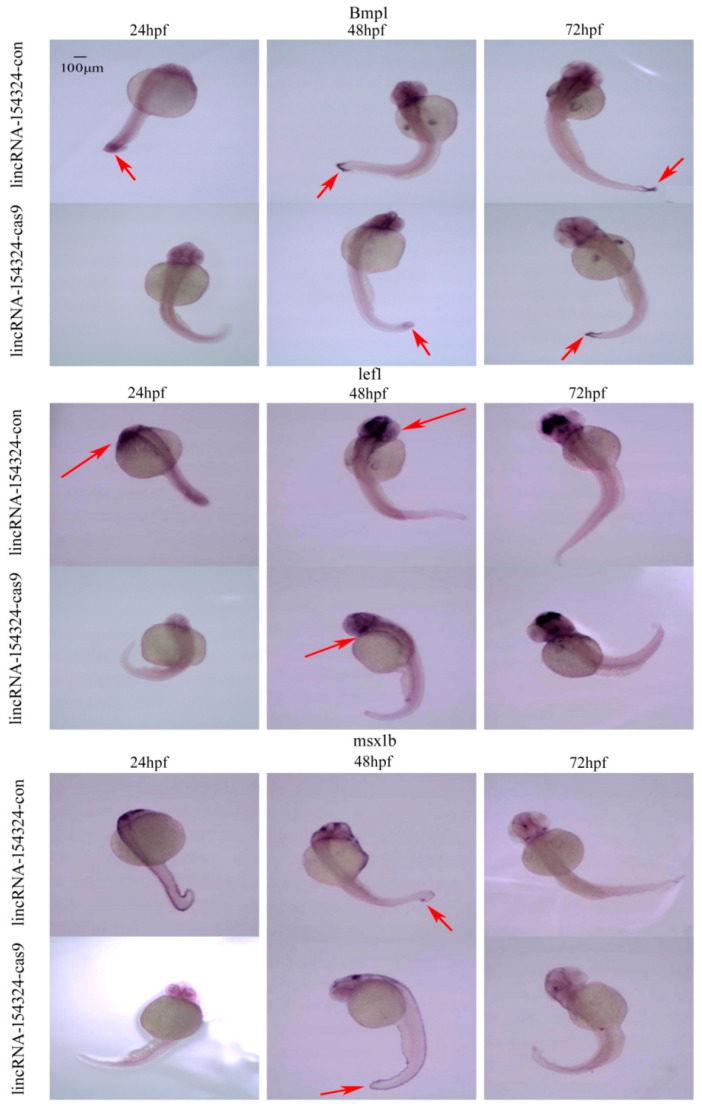
The temporal and spatial expression of marker genes related to caudal fin regeneration in 24–72 hpf embryos after lincRNA-154324 gene knockout. *Bmp1a*, *lef1*, and *msx1b* spatial expression in the lincRNA-154324-con and lincRNA-154324-cas9 groups at different stages of zebrafish embryogenesis. Red arrows: pointing to the site of gene expression. Scale bars: 100 μm.

**Figure 15:**
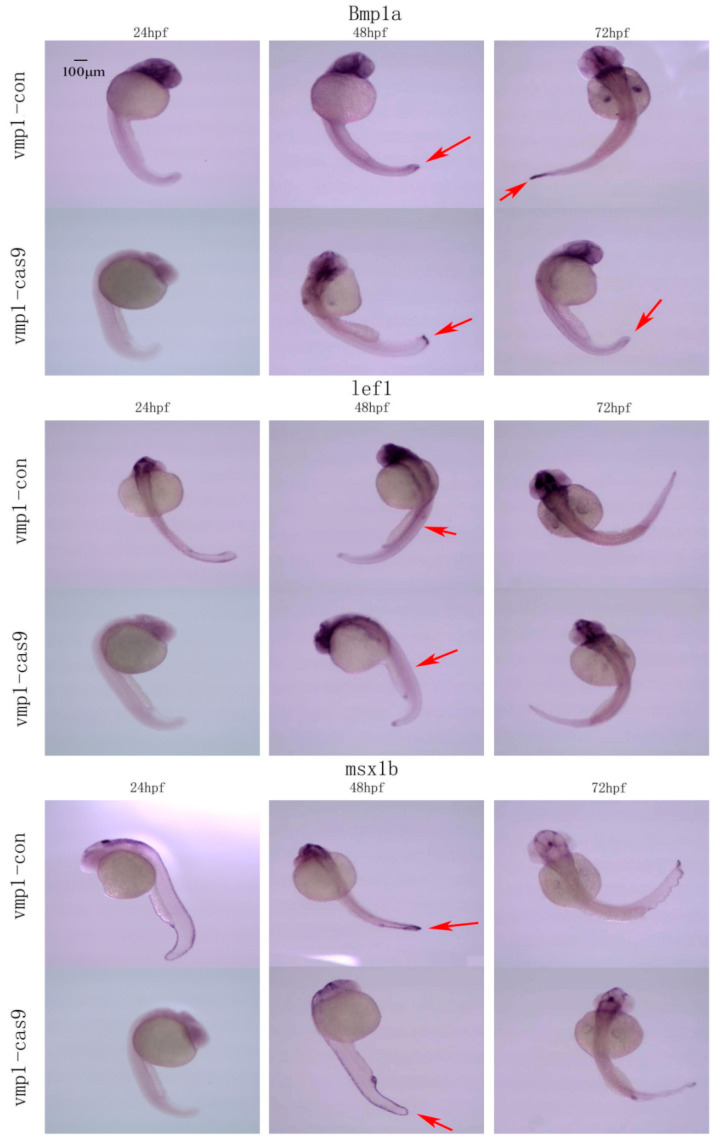
The temporal and spatial expression of marker genes related to caudal fin regeneration in 24–72 hpf embryos following *vmp1* gene knockout. *Bmp1a*, *lef1*, and *msx1b* spatial expression in the *vmp1*-con and *vmp1*-cas9 groups at different stages of zebrafish embryogenesis. Red arrows: pointing to the site of gene expression. Scale bars: 100 μm.

After the CRISPR/Cas9 knockout micro-injection of the zebrafish embryos, WISH was conducted to detect the expression levels of three marker genes—bone morphogenetic protein (*Bmp1a*), lymphoid-enhancer-binding-factor (*lef1*), and muscle segment bomeobox 1b (*msx1b*)—of caudal fin regeneration at 24, 48, and 72 h post-fertilization (hpf), respectively. After lincRNA-154324 was knocked out, the expression levels of *Bmp1a*, *lef1*, and *msx1b* were lower than those in the control group from 24 to 72 hpf, indicating that lincRNA-154324 knockout suppressed the expression of caudal fin regeneration-related marker genes. Similarly, the expression levels of *Bmp1a*, *lef1*, and *msx1b* were lower than those in the control group from 24 to 72 hpf following *vmp1* knockout, indicating that *vmp1* knockout suppressed the expression of caudal fin regeneration-related marker genes (Figure 14 and Figure 15).

## 3. Discussion

Regeneration has always been a focus and hotspot of scientific research. Zebrafish caudal fins are widely used in tissue regeneration research because of their strong regenerative ability and the existence of similar gene regulation pathways in humans [29]. Caudal fin regeneration is an extremely complex process in which a variety of molecular and biological phenomena, such as cell proliferation, apoptosis, migration, and differentiation, are involved [30,31,32,33]. This process is precisely regulated by a variety of gene regulatory networks and multiple signaling pathways. However, many questions regarding caudal fin regeneration in zebrafish are still unanswered. Previous studies on caudal fin regeneration in zebrafish have mostly focused on coding genes [34]. Little attention has been paid to ncRNAs, especially lncRNAs. In order to comprehensively explore the expression of lncRNAs during caudal fin regeneration in zebrafish, this study used the RNA-seq method to analyze the expression of lncRNAs in the newly regenerated caudal fin at 3 and 7 dpa, using the caudal fin at 0 dpa as the control group. RNA-seq was conducted to screen out the DE lncRNAs in the regenerated caudal fins during the critical period. Our study clearly demonstrated that lncRNAs are differentially expressed during zebrafish caudal fin regeneration, especially at 3 dpa. This result suggests that lncRNAs may play a role in the early stage of caudal fin regeneration (3 dpa).

The biological processes and signaling pathways involved with DE lncRNAs in zebrafish caudal regeneration are still not well-defined. Here, we employed two fundamental types of bioinformatics analysis for the definition of DE lncRNAs, GO and KEGG. GO is an internationally standardized transcript functional classification system. The system provides a complete set of standard vocabularies describing the properties of transcripts in the body, which are used to clarify the biological processes, molecular functions, and cellular components involved in transcripts and transcript-related products in the body. KEGG is the main public database related to pathways, and KEGG pathway analysis of differentially expressed genes can accurately locate the signal pathways they participate in. In order to gain further understanding of the specific roles of these DE lncRNAs in the process of caudal fin regeneration, GO and KEGG pathway enrichment analyses were performed for the target genes of DE lncRNAs (cis target genes of). The results revealed that a large number of DE lncRNAs are related to classic signaling pathways and various biological processes, some of which are deeply implicated in the regenerative process. This finding suggests that DE lncRNA may regulate caudal fin regeneration through classical biological processes and signaling pathways of caudal fin regeneration. Moreover, it provides many clues for the exploration of the specific mechanism of lncRNA in caudal fin regeneration.

In the present study, RNA-seq was first used to identify the DE lncRNAs involved in the process of caudal fin regeneration in zebrafish. The verification of the genes closely related to caudal fin regeneration from among a large number of DE lncRNAs was the next key issue. Unlike mRNAs, lncRNAs have low conservation, no open reading frames, and their regulatory mechanisms are complex and changeable. Recent studies indicate that lncRNAs can regulate gene expression through a variety of mechanisms, which can be summarized as epigenetic regulation, transcription regulation, and post-transcriptional regulation. However, there have been inconsistencies in the results obtained in lncRNA studies in recent years [35]. Therefore, in this study, lncRNA screening could not only rely on the literature, and bioinformatics and other in-depth analyses, such as temporal and spatial expression, were used to screen out the lncRNAs that may be related to zebrafish regeneration. We verified the results of the RNA-seq using RT-qPCR analysis for the DE lncRNAs. The expression patterns showed similar results to those obtained with the RNA-seq (Figure 6), which indicated that lncRNAs might play a role in caudal fin regeneration.

Currently, there are no expression studies of lncRNAs involved in the caudal fin regeneration process in zebrafish. Based on the above results, we selected the DEG lincRNA-154324, which is related to caudal fin regeneration and co-expressed between groups, and conducted in-depth research on it. We identified a pair of cis genes (lincRNA-154324 and *vmp1*) based on the analysis of the correlation between lncRNAs and mRNAs in the RNA-seq data. After biological analysis of this pair of cis genes, it was found that the correlation reached more than 0.8, and the expression pattern, which was almost the same as the expression trend for RNA-seq, was verified with RT-qPCR (Figure 7B,D). Interestingly, lincRNA-154324 was up-regulated in the pro-regenerative macrophage subtype during heart regeneration in zebrafish, which led us to wonder whether it might be involved in the regeneration of the zebrafish caudal fin [36]. Bioinformatics analysis showed that lincRNA-154324 is about 916 bp in length and located on zebrafish chromosome 15 (chromosome 15: 17,376,367–17,378,063), and it has six exon sequences. According to the protein-coding gene classification, it is an lncRNA. The transcript of the adjacent protein coding gene *vmp1* is about 2135 bp in length and is located on zebrafish chromosome 15 (chromosome 15: 17,343,319–17,373,352), with 12 exons. Studies have reported that over-expression of *vmp1* can inhibit pro-apoptotic signal transduction by increasing the turnover rate of dysfunctional mitochondria, thereby inhibiting apoptosis [37]. In addition, studies have shown that *vmp1* is essential for the initial cell–cell contact and tight junction process, and its expression level is related to the invasion and metastasis potential of cancer cells. The expression of *vmp1* in colon cancer tissues was significantly lower than that in neighboring non-cancerous tissues and was negatively correlated with the malignant degree of cancer; that is, the down-regulation of the expression of *vmp1* could reduce cell adhesion and invasion ability [38,39,40]. At the same time, it was found that *vmp1* and miRNA-21 could regulate each other near the downstream region, and *vmp1* might play an important role in tumor proliferation, invasion, and metastasis [41]. Interestingly, in this study, we found that the lincRNA-154324 was positively correlated with the cis-neighboring gene *vmp1*. Importantly, the cis correlation coefficient between lincRNA-154324 and *vmp1* was extremely high. The region downstream of *vmp1* includes miRNA-21, which is not only initiated by itself but also regulated by *vmp1* promoter. Therefore, lincRNA-154324 and miRNA-21were not only related to cis genes, but also located downstream of *vmp1*. We speculate that the expression of *vmp1* may be inextricably related to its neighboring non-coding RNA (lincRNA-154324 and miRNA-21). If so, this would provide a certain experimental and theoretical basis for the future study of the regulatory role of lincRNA-154324 in caudal fin regeneration.

Analyzing the characteristics of the temporal and spatial expression of selected genes can also provide us with a certain basis for screening the target gene. In order to reveal the relation between lincRNA-154324 and *vmp1* during caudal fin regeneration, we conducted WISH to detect their expression. The WISH results showed that lincRNA-154324 was weakly expressed in the wound epidermis at 1 dpa and the expression then gradually increased, reaching the highest level at 3 dpa. The expression trend of *vmp1* was almost the same as that of lincRNA-154324. It was particularly noteworthy that both genes were initially expressed in the wound epidermis and then gradually transferred to the vicinity of the blastema and blood vessels. This result indicates that these two genes might play important roles in caudal fin regeneration.

Currently, there are no functional studies of the role of lincRNA-154324 in the caudal fin regeneration process in zebrafish. In this study, for the first time, we successfully knocked out the expression of lincRNA-154324 and *vmp1* in the regeneration of zebrafish caudal fins via CRISPR/Cas9 technology. We found that lincRNA-154324 and *vmp1* are essential for the process of caudal fin regeneration. It was also found that embryonic development in the knockout group was slower than in the control group. Meanwhile, the statistical results showed that either lincRNA-154324 or *vmp1* knockout greatly increased both the death rate and malformation rate of the zebrafish embryos, suggesting that the two genes may play important roles in the embryonic development of zebrafish. At 7 dpf, morphological observation of zebrafish embryos after CRISPR/Cas9 knockout injection showed that the embryos in the zebrafish knockout group had delayed development, shortened body lengths, pericardial edema, curved spines, curled-up caudal fins, and anterior and posterior truncations, and some even had no caudal parts. These phenotypes indicated that gene knockout caused developmental abnormalities, implying that candidate genes may play roles in zebrafish embryonic development. The caudal fin curling up and the tailless fish also illustrated that these genes have important roles in the growth and development of the caudal fin.

*Bmp1a*, *lef1*, and *msx1b*, as important marker genes in caudal fin regeneration, play important roles in fin formation, blastema tissue formation and cell proliferation in caudal fin regeneration. Therefore, WISH was used to detect the spatiotemporal expression of caudal fin regeneration-related marker genes in 24, 48, and 72 hpf embryos following knockout. The results showed that the expression levels of the caudal fin regeneration-related marker genes *Bmp1a*, *lef1* and *msx1b* were lower than those in the control group in 24–72 hpf embryos following lincRNA154324 gene knockout (Figure 14). After *vmp1* gene knockout, the expression levels of the caudal fin regeneration-related marker genes *Bmp1a*, *lef1*, and *msx1b* were lower than those in the control group in 24–72 hpf embryos (Figure 15). Therefore, the lower expression of caudal fin regeneration marker genes in the gene knockout group again confirms that lincRNA-154324 and *vmp1* play important roles in caudal fin regeneration in zebrafish, but the underlining mechanism still needs to be addressed.

## 4. Materials and Methods

### 4.1. Animal and Fin Amputation

The AB wild-type zebrafish used in this study (12 month old males and females with an average weight of 0.3 g) were purchased from Wuhan Zebrafish Resource Center, China. The experimental fish were reared in the standard zebrafish breeding system of our laboratory, with a photoperiod of 14 h (08:30–22:30) light and 10 h (22:30–08:30) dark in a closed loop aquaculture system. They were fed twice a day with live artemia that had just been hatched. The sexually mature male and female zebrafish were crossed and the fertilized eggs produced in the corresponding time period were collected. The adult fish were first anesthetized with 0.1% tricaine, and then about 50% of the fins were cut off with ophthalmic scissors. The fish were cultured in 28 °C water until the regenerated caudal fins were collected for subsequent experiments during the corresponding period of time. The fish were handled according to the guidelines in the China Law for Animal Health Protection and Instructions for Granting Permits for Animal Experimentation for Scientific Purposes (ethics approval no. SCXK (YU) 2005–0001).

### 4.2. Total RNA Isolation and Whole Transcriptome Sequencing

Three time points—i.e., 0, 3, and 7 days post-amputation (dpa)—during the zebrafish caudal fin regeneration process were selected, and each time period was investigated three times for a total of nine samples (each sample included 20 caudal fins, and the 20 caudal fins were mixed together). The caudal fin tissue regenerated in the corresponding time period was collected for total RNA isolation using the Trizol method. The quality of RNA was detected with the following three methods: (1) agarose gel electrophoresis detection, (2) NanoDrop micro-spectrophotometer detection, and (3) Agilent 2100 detection. The whole RNA-seq and bioinformatics analyses were performed by Gene Denovo Biotechnology Co. (Guangzhou, China). In brief, after the total RNA was extracted, the built sequencing library was sequenced using an Illumina HiSeqTM 4000. The clean reads of each sample were then mapped to GRCz10 in Ensembl-Release91 using TopHat2 version 2.0.3.12 [42]. Transcript abundances were quantified using RSEM software. The transcript expression level was normalized using the fragments per kilobase of transcript per million mapped reads (FPKM) method, With 10G sequencing per sample. The differentially expressed genes (DEGs) of the lncRNAs and mRNAs were analyzed using the edgeR package (http://www.r-project.org/) (accessed on 1 September 2019). The DEGs of lncRNAs and mRNAs were calculated with a fold change ≥ 2 or ≥1 and *p* < 0.05 or FDR < 0.05 as thresholds. The analysis of the correlation between lncRNAs and mRNAs included antisense analysis, cis analysis, and trans effect analysis. The basic principle of cis-acting target gene prediction is that the function of an lncRNA is related to its neighboring protein-coding genes. The upstream lncRNA may overlap with the promoter or other cis-acting elements of the co-expressed gene, which can affect the gene expression at the transcriptional or post-transcriptional level. The lncRNA located at the 3’UTR or downstream of the gene may be involved in other regulatory effects. Therefore, we annotated the lncRNAs that were found to be located in the “unknown region” in the previous analysis. If they were located within 10 kb upstream or downstream of a gene, these lncRNAs could overlap with the region where the cis-acting element was located, thereby participating in the process of transcriptional regulation.

All the transcriptome datasets in this study are available at the National Center for Biotechnology Information Gene Expression Omnibus under the accession number GSE160909.

### 4.3. The Enrichment Analysis of GO and KEGG

The Gene Ontology (GO) and Kyoto Encyclopedia of Genes and Genomes (KEGG) enrichment analyses were conducted for DEGs using the GO database (http://www.geneontology.org/) (accessed on 19 September 2019) and the KEGG database, respectively. GO and KEGG results with *p* ≤ 0.05 or FDR ≤ 0.05 were defined as GO (KEGG) terms that were significantly rich in DEGs.

### 4.4. cDNA Synthesis and RT-qPCR

When verifying the gene expression at the corresponding stage (0, 3, 7 dpa) of caudal fin regeneration in zebrafish, the regenerated caudal fins of 30 fish were amputated at each stage and randomly divided into three groups (10 fish/group, three repeats/group) for RNA extraction. The total RNA in the regenerated fin tissue at the corresponding stage was extracted using Trizol reagent (TaKaRa, Kyoto, Japan). The quality and concentration of RNA were evaluated with agarose-gel electrophoresis and NanoDrop One, respectively. A cDNA synthesis kit (Roche, Basel, Switzerland) was used to produce the first-strand cDNA, and 1 μg of total RNA was used for each reaction. RT-qPCR was performed in q225 (Kubo, Beijing, China) with SYBR Green qPCR Mix (Monad, Zhuhai, China). The primers needed in this study were all designed online by Primer3Plus, and the primers were synthesized by GENEWIZ Biotechnology Co., Ltd. The primer names and sequence information are shown in Table 2.

### 4.5. Whole-Mount In Situ Hybridization

Whole-mount in situ hybridization (WISH) was conducted to detect the temporal and spatial expression of the regeneration-related genes during the process of caudal fin regeneration. As described in our previous experiment, WISH was performed with embryos or regenerated caudal fins at the corresponding time points [43]. Probes for WISH were prepared. On the first day of WISH, the collected tissues were quickly rehydrated and digested with proteinase K, and the tissues were randomly fixed and the digestion terminated. Finally, the tissues were washed and placed in a 70 °C water bath in a hybridization solution containing RNA probes (1ng/l) for 14 h. On the second day of WISH, the tissue was washed and blocked in blocking solution for 2–3 h. Finally, a blocking solution containing DIG was added to the tissue, and the samples were left overnight at 4 °C. On the third day of WISH, the tissues were washed and stained in staining solution and finally imaged. Each experiment was performed in triplicate (ten replicates for each sample of embryos in different time periods; the caudal fins regenerated in different time periods had three biological replicates for each sample). The WISH probe primer names and sequence information are shown in Table 3.

### 4.6. CRISPR/Cas9-SgRNA Micro-Injection

The CRISPR/Cas9 target was designed on the website https://www.crisprscan.org/?page=sequence (accessed on 1 September 2020) (Table 3). SgRNA in vitro transcription was performed in a one-step method, and the SgRNA-guides sequence, synthesis, and transcription system were as shown in the kit instructions (Novoprotein, Shanghai, China). The one-step transcribed SgRNA and Cas9-nuclease (Novoprotein, Shanghai, China) were assembled and then subjected to embryo micro-injection. A total of about 280 embryos (at the one-cell stage) produced by a pair of fish were used for micro-injection of CRISPR-Cas9 knockout-related genes, and 280 embryos with the same developmental stage without any treatment were used as a controls group. Injection was performed with a pneumatic embryo micro-injector (ZGEENBIO, Taiwan, China). Embryos from more than one pair of fish were used to ensure the viability of embryo batches. Zebrafish embryo development were roughly divided into eight stages, including the zygotic stage, cleavage stage, blastocyst stage, gastrulation stage, staging stage, pharyngeal sac stage, hatching stage, and early juvenile stage [44]. The knockout micro-injection was performed as far as possible into the one-cell stage of the zygote stage.

### 4.7. Statistical Analysis

SPSS 22.0 statistical software was used for data analysis. In accordance with the different experiments, the data were analyzed with one-way ANOVA and represented as mean ± standard error of mean (SEM). Multiple comparisons between the groups were performed using the S-N-K method. Values of *p* < 0.05 between the treatment group and control group were considered statistically significant (* *p* < 0.05, ** *p* < 0.01). At least three biological replicates were performed in the experiment.

## 5. Conclusions

In summary, this study screened the differently expressed lncRNAs in three stages of zebrafish caudal fin regeneration using RNA-seq. Moreover, the results for the temporal and spatial expression and the function analysis for lincRNA-154324 and *vmp1* showed that lncRNA might have important regulatory roles in the growth and development of the caudal fin. However, the specific mechanism of lncRNA during caudal fin regeneration still needs to be revealed in future studies.

## Figures and Tables

**Table 1:** Summary of lncRNA-seq for each sample.

Sample	HQ Clean Data (bp)	Q20 (%)	Q30 (%)	N (%)	GC (%)
CK0dpa-1	12,363,583,806	12,148,867,942 (98.26%)	11,704,232,201 (94.67%)	97,831 (0.00%)	6,493,011,448 (52.52%)
CK0dpa-2	13,077,526,836	12,864,728,601 (98.37%)	12,412,532,979 (94.91%)	102,739 (0.00%)	6,728,058,609 (51.45%)
CK0dpa-3	12,836,297,773	12,622,712,157 (98.34%)	12,171,651,134 (94.82%)	101,103 (0.00%)	6,576,887,421 (51.24%)
T3dpa-1	13,050,013,929	12,830,474,508 (98.32%)	12,370,609,292 (94.79%)	103,729 (0.00%)	6,786,784,748 (52.01%)
T3dpa-2	12,507,277,837	12,287,134,445 (98.24%)	11,830,170,653 (94.59%)	98,548 (0.00%)	6,525,662,021 (52.17%)
T3dpa-3	12,225,132,989	12,006,382,727 (98.21%)	11,553,838,554 (94.51%)	96,100 (0.00%)	6,340,284,367 (51.86%)
T7dpa-1	12,293,254,313	12,069,636,216 (98.18%)	11,596,123,006 (94.33%)	99,404 (0.00%)	6,556,408,680 (53.33%)
T7dpa-2	12,505,709,229	12,291,214,684 (98.28%)	11,843,282,011 (94.70%)	98,982 (0.00%)	6,592,244,203 (52.71%)
T7dpa-3	11,194,015,091	10,956,042,369 (97.87%)	10,508,065,794 (93.87%)	1,033,153 (0.01%)	5,906,056,545 (52.76%)

**Table 2:** The sequences of primers used in this study.

Primers	Primer Sequences (5′→3′)
β-actin-F	TTCACCACCACAGCCGAAAGA
β-actin-R	TACCGCAAGATTCCATACCCA
ENSDART00000148196-qPCR-F	GTCTGGACTGAGTGAAATCT
ENSDART00000148196-qPCR-R	TTACAGCAGGTACAGTATGG
ENSDART00000155541-qPCR-F	CCTCTGATAACTTCTTAGGC
ENSDART00000155541-qPCR-R	AGGAGCTGAACTCATCTTAG
ENSDART00000175059-qPCR-F	AGAAGAGCTACTGTGAGAGC
ENSDART00000175059-qPCR-R	AGAGGTCCAGTTAGACAGAC
ENSDART00000176229-qPCR-F	ATCAAGAGCAGTAGTCCAGT
ENSDART00000176229-qPCR-R	TCTGTAGCTGTGTTATCCTG
ENSDART00000176871-qPCR-F	GAAGAAGGCTTTCTACAGTG
ENSDART00000176871-qPCR-R	GTCTGTGTTAACATCCAGTG
ENSDART00000177366-qPCR-F	GTTCTCAACAAAGAGGTAGG
ENSDART00000177366-qPCR-R	GGTTTCTGTGAGTTACAGGT
ENSDART00000177369-qPCR-F	GGTTTCCTGAACAGAGACTA
ENSDART00000177369-qPCR-R	GAATACAACCTAACCAGTGC
ENSDART00000177544-qPCR-F	GACGTTGAGGCTGTTTAG
ENSDART00000177544-qPCR-R	CTGATCTGTCATTCTGTCTG
ENSDART00000179287-qPCR-F	GTTCAACCTAGAAGGTCATC
ENSDART00000179287-qPCR-R	CTTAAGCCACAGTATGTCTG
ENSDART00000174415-qPCR-F	GAGAGAGCAGATTCAATGTC
ENSDART00000174415-qPCR-R	GATCATTGAGAGACGAGACT
ENSDART00000174069-qPCR-F	GAGACTCCACACTTCTGAAT
ENSDART00000174069-qPCR-R	TGTATCTCTAGTGGCTGATG
ENSDART00000158007-qPCR-F	GAGAGTGAGCAGTCAAAAAC
ENSDART00000158007-qPCR-R	GACCTACAAAATCTGAGGAG
ENSDART00000007434-qPCR-F	GAGAAACACATCCTGAAGAC
ENSDART00000007434-qPCR-R	TATCTCTGATGTAGCGACTG
*Vmp1*-qPCR-F	ATGGAAGCTTTGGCAGAGAA
*Vmp1*-qPCR-R	TACCCAGTAGGCACACCACA
ENSDART00000154324-qPCR-F	CACACCAGAGAACCTGCTGA
ENSDART00000154324-qPCR-R	TGCTGCTGAAACCACTCATC

**Table 3:** The sequences of the probes and CRISPR/Cas9-sgRNA primers used in this study.

Primers	Primer Sequences (5′→3′)
*Vmp1*-probe-F	TGTGGTGTGCCTACTGGGTA
*Vmp1*-probe-R	CAGAGACACCATCTGCTCCA
ENSDART00000154324-probe-F	TCAAAGGAAGAGGACGCAGT
ENSDART00000154324-probe-R	TCAGCAGGTTCTCTGGTGTG
*Bmp1a*-probe-F	TTGGTACGATCACGTGGAAA
*Bmp1a*-probe-R	GTTGTCGGGTCTGGAACACT
*lef1*-probe-F	TCCCAGAACGTCGAATAAGG
*lef1*-probe-R	GGCCGAGGATCTGATTGATA
*msx1b*-probe-F	GACCCGTTGAAACGACATCT
*msx1b*-probe-R	GTGAGGTTGAGGGAGTTGGA
*vmp1*-sgRNA-6wxz	TTAATACGACTCACTATAGGGCTGGATACGGCGGCTCGGTTTTAGAGCTAGAAATAGCA
lincRNA-154324-sgRNA-2wxz	TTAATACGACTCACTATAGGCCGATTGCTTGGACGAGGGTTTTAGAGCTAGAAATAGCA
*vmp1*-f0jc-6wxz-F	ACCACCAATCCAATCCTG
*vmp1*-f0jc-6wxz-R	AGTTTCTACTCCCACCAG
lincRNA-154324-f0jc-2wxz-F	AAAGACCTGCCAAGCCATCG
lincRNA-154324-f0jc-2wxz-R	CAGCAGGTTCTCTGGTGTGA

## Data Availability

Represented data are publicly archived datasets. For further information, please contact the corresponding author.

## Supplementary Materials

The following supporting information can be downloaded at: https://www.mdpi.com/article/10.3390/ijms23168944/s1.
