# Supplementary material for: The Expression and Function of lincRNA-154324 and the Adjoining Protein-Coding Gene vmp1 in the Caudal Fin Regeneration of Zebrafish
Source: Int J Mol Sci. 2022 Aug 11;23(16):8944. doi: 10.3390/ijms23168944 (PMC9409064; doi:10.3390/ijms23168944)
Supplement: Supplementary file 1 [file ijms-23-08944-s001.zip › ijms-1862899-supplementary.pdf]

Supplemental table

Table S1

GeneID	CK0dpa_fpkm	T3dpa_fpkm	log2(FC)	Pvalue	FDR	significant	Symbol
ENSDART00000007434	6.01	0.813333333	-2.885446344	7.23E-15	2.28E-12	yes	BX005421.1
ENSDART00000059613	1.743333333	0.51	-1.773279294	0.024177164	0.20403566	no	BX005421.2
ENSDART00000082740	1.65	4.303333333	1.38298857	6.61E-09	5.90E-07	yes	CT573494.2
ENSDART00000087449	0.096666667	0.98	3.34169135	6.56E-13	1.37E-10	yes	CR847968.1
ENSDART00000133496	7.163333333	2.18	-1.716302942	0.001506597	0.026310396	yes	CU467110.1
ENSDART00000136012	0.743333333	2.016666667	1.439891432	4.58E-08	3.09E-06	yes	CR388373.2
ENSDART00000137680	0.023333333	0.433333333	4.215012891	7.64E-06	0.000347058	yes	BX663503.1
ENSDART00000139663	0.443333333	1.24	1.483876376	0.041239933	0.290775041	no	CR391910.2
ENSDART00000141092	5.733333333	2.176666667	-1.397253668	0.03023909	0.23980078	no	BX469930.1
ENSDART00000142561	1.486666667	0.093333333	-3.993544978	0.000999383	0.018630294	yes	BX323590.2
ENSDART00000143303	2.286666667	0.86	-1.410837511	0.019547572	0.177855236	no	BX323590.2
ENSDART00000144687	6.893333333	0.863333333	-2.997212183	9.47E-09	7.54E-07	yes	CABZ01015815.1
ENSDART00000145442	0.176666667	0.56	1.664396968	0.041439517	0.290775041	no	CR936321.1
ENSDART00000145963	0.853333333	1.82	1.092757141	0.000279348	0.006978113	yes	CR391990.1
ENSDART00000148196	25.91	5.576666667	-2.216034071	2.10E-07	1.25E-05	yes	AL929237.1
ENSDART00000148329	1.373333333	0.306666667	-2.162938571	0.000672389	0.013780918	yes	BX088709.1
ENSDART00000150088	0.07	1.51	4.431049817	3.77E-05	0.001359578	yes	BX649485.2
ENSDART00000150683	1.863333333	0.48	-1.956779471	0.001489114	0.026195815	yes	mhc1zda
ENSDART00000151148	0.363333333	0.001	-8.505149919	0.036033808	0.264134315	no	BX957317.2
ENSDART00000151887	0.323333333	1.683333333	2.380226735	0.000334393	0.008031868	yes	BX901907.5
ENSDART00000153157	0.001	0.253333333	7.984893108	0.046252695	0.31311445	no	CR749163.1
ENSDART00000153298	10.13	36.55666667	1.851500354	7.29E-15	2.28E-12	yes	AL732488.1
ENSDART00000153366	0.06	1.006666667	4.068479738	0.00063544	0.013338908	yes	CR382372.1
ENSDART00000153475	1.96	3.943333333	1.008562014	1.48E-05	0.000607842	yes	CU467861.1
ENSDART00000153479	0.37	0.01	-5.209453366	0.006328905	0.083648707	no	CR377211.3
ENSDART00000153498	0.25	1.026666667	2.03796785	0.000293568	0.007260716	yes	BX294100.1
ENSDART00000153691	1.443333333	3.803333333	1.397859862	3.01E-05	0.001121817	yes	CU928129.1
ENSDART00000153803	0.416666667	1.843333333	2.145351386	2.89E-06	0.000144153	yes	CU466287.2
ENSDART00000154176	58.17	22.69333333	-1.358006768	0.026731243	0.2172617	no	BX537305.1
ENSDART00000154290	0.033333333	0.223333333	2.744161096	0.034003749	0.255079175	no	CU972512.1
ENSDART00000154324	0.09	1.006666667	3.483517237	8.20E-06	0.000365895	yes	CR848791.3
ENSDART00000154910	3.796666667	0.683333333	-2.474071932	3.81E-05	0.001359578	yes	BX005421.1
ENSDART00000155099	0.94	0.116666667	-3.010268335	0.01957974	0.177855236	no	CR354430.3
ENSDART00000155205	0.001	2.983333333	11.54270947	1.63E-10	1.93E-08	yes	CU927934.1
ENSDART00000155306	2.51	8.25	1.716706755	7.93E-07	4.40E-05	yes	CR925728.1
ENSDART00000155541	69.42666667	5.64	-3.621722838	2.47E-09	2.37E-07	yes	CU693494.2

ENSDART00000155766	0.06	0.186666667	1.637429921	0.039526831	0.282108636	no	BX957317.2
ENSDART00000155782	0.33	0.001	-8.366322214	0.016239693	0.163500813	no	CU693494.3
ENSDART00000155800	0.54	1.273333333	1.237578825	0.008453823	0.101244796	no	CR759845.1
ENSDART00000155813	0.001	0.283333333	8.14635653	0.000894671	0.017060221	yes	CU927934.3
ENSDART00000155964	0.013333333	0.186666667	3.807354922	0.015618976	0.159902462	no	CR628341.1
ENSDART00000156268	1.96	3.943333333	1.008562014	1.48E-05	0.000607842	yes	CU467861.1
ENSDART00000156279	0.983333333	1.99	1.017015977	0.000606723	0.012844017	yes	BX890558.1
ENSDART00000156294	4.79	1.303333333	-1.877819549	0.00057497	0.012598906	yes	CR848667.1
ENSDART00000156448	0.113333333	1.096666667	3.274480932	9.28E-05	0.002727767	yes	zgc:194207
ENSDART00000156913	0.133333333	0.43	1.689299161	0.001907711	0.032418116	yes	BX927184.1
ENSDART00000158002	0.01	0.206666667	4.36923381	0.001846484	0.031592579	yes	CR812832.1
ENSDART00000158007	13.52	2.066666667	-2.709717532	7.19E-10	7.34E-08	yes	CU467961.1
ENSDART00000158564	14.04333333	4.716666667	-1.574045863	0.000443148	0.010107892	yes	CU681842.1
ENSDART00000158696	1.16	0.013333333	-6.442943496	2.94E-06	0.000144153	yes	mhc1zda
ENSDART00000160090	41.98666667	19.04666667	-1.140392721	0.021821393	0.189270278	no	BX901923.2
ENSDART00000160098	2.426666667	0.076666667	-4.984232684	2.35E-06	0.000119742	yes	BX901923.2
ENSDART00000161094	0.596666667	0.001	-9.220781371	0.036403395	0.265334294	no	BX640547.1
ENSDART00000161509	2.44	0.516666667	-2.239575433	0.049673297	0.328651649	no	BX936433.1
ENSDART00000161583	0.266666667	0.94	1.817623258	1.67E-11	2.55E-09	yes	FP102783.1
ENSDART00000161755	7.676666667	2.836666667	-1.436283374	0.033611428	0.253811668	no	CU681842.1
ENSDART00000162136	1.203333333	0.083333333	-3.851998837	0.01902035	0.176028966	no	FP016106.1
ENSDART00000162258	6.85	2.373333333	-1.529189248	0.011818739	0.128922316	no	BX901957.1
ENSDART00000162659	0.353333333	0.943333333	1.416737788	0.046696751	0.31526617	no	CU856423.1
ENSDART00000163057	9.71	3.503333333	-1.470743032	0.00169063	0.029125473	yes	FP101882.1
ENSDART00000163410	11.26333333	4.06	-1.472082217	0.009359492	0.109566911	no	CU468917.1
ENSDART00000163592	0.001	0.153333333	7.26052755	0.017491295	0.170564923	no	AL645792.2
ENSDART00000164487	0.01	0.123333333	3.624490865	0.047291609	0.318421669	no	FP565451.1
ENSDART00000164681	0.001	0.223333333	7.803054785	0.043490117	0.299279098	no	FP016142.2
ENSDART00000167160	1.236666667	0.283333333	-2.12588444	0.011072194	0.122925953	no	BX927122.1
ENSDART00000168210	3.493333333	1.533333333	-1.187932951	0.03281669	0.2514604	no	FP016106.1
ENSDART00000169453	4.34	13.86	1.67516031	1.72E-12	3.07E-10	yes	CU469577.1
ENSDART00000172265	1.713333333	0.313333333	-2.451035698	0.007098564	0.09187675	no	CU639397.1
ENSDART00000172918	0.246666667	0.733333333	1.571906348	0.017083556	0.168010717	no	BX005228.1
ENSDART00000173479	0.946666667	2.213333333	1.225292312	1.07E-06	5.69E-05	yes	CR388231.2
ENSDART00000173489	2.43	0.52	-2.224372785	0.044536144	0.304798047	no	BX957297.2
ENSDART00000173632	0.001	0.576666667	9.171593822	0.001516692	0.026310396	yes	FO704741.1
ENSDART00000173718	13.90666667	5.64	-1.302009589	0.015166069	0.15590469	no	CR388099.1
ENSDART00000174069	12.06666667	1.13	-3.416632519	3.35E-17	2.09E-14	yes	CABZ01015815.1
ENSDART00000174163	0.283333333	1.373333333	2.277109591	0.00044915	0.010107892	yes	CABZ01068273.1
ENSDART00000174365	101.8566667	38.23333333	-1.413637519	0.009616603	0.110193915	no	CABZ010705

							27.1
ENSDART00000174374	69.12666667	28.21666667	-1.292694792	0.041414474	0.290775041	no	CABZ01070527.1
ENSDART00000174415	14.76333333	4.23	-1.803288928	0.000305981	0.007493538	yes	CR376839.1
ENSDART00000174417	0.02	0.303333333	3.922832139	0.003138419	0.047803479	yes	AL928927.2
ENSDART00000174479	0.11	0.32	1.540568381	0.005009304	0.069560803	no	CABZ01077124.2
ENSDART00000174664	0.001	0.11	6.781359714	0.00470034	0.067093997	no	FO704589.1
ENSDART00000174725	0.001	0.146666667	7.196397213	0.016293899	0.163500813	no	CABZ01084134.1
ENSDART00000174729	4.17	1.37	-1.60587149	0.023957647	0.20286848	no	BX255935.2
ENSDART00000174739	0.586666667	0.106666667	-2.459431619	0.026500794	0.217045843	no	CABZ01002339.2
ENSDART00000174837	0.723333333	0.001	-9.498516827	0.019907539	0.178881409	no	CABZ01012866.1
ENSDART00000174873	0.756666667	0.19	-1.993658473	0.004258894	0.061853012	no	CABZ01004903.1
ENSDART00000174913	0.236666667	0.001	-7.886712714	0.041884117	0.291836878	no	CABZ01088053.1
ENSDART00000174956	8.856666667	2.693333333	-1.71737103	0.000248829	0.006342609	yes	BX927329.2
ENSDART00000174983	5.653333333	1.163333333	-2.280837228	1.09E-05	0.000462391	yes	CABZ01077121.1
ENSDART00000174990	0.146666667	0.35	1.254813899	0.026117976	0.214614162	no	CABZ01048956.2
ENSDART00000175018	0.233333333	0.65	1.478047297	0.000223294	0.0058103	yes	CABZ01007102.1
ENSDART00000175059	3.99	0.62	-2.686048626	7.35E-10	7.34E-08	yes	BX571711.3
ENSDART00000175077	0.001	0.413333333	8.691161905	0.000602256	0.012844017	yes	CABZ01079894.1
ENSDART00000175105	1.093333333	0.376666667	-1.537373042	0.023795966	0.202663252	no	CR450695.1
ENSDART00000175123	0.22	0.001	-7.781359714	0.025613639	0.211587856	no	CR925768.3
ENSDART00000175130	0.373333333	0.043333333	-3.106915204	0.016639123	0.165544368	no	CABZ01059413.1
ENSDART00000175143	1.353333333	0.313333333	-2.110747066	0.020253237	0.180687807	no	BX649411.3
ENSDART00000175228	1.063333333	0.13	-3.032010395	0.022124602	0.189921837	no	CU462979.1
ENSDART00000175255	2.8	7.543333333	1.429775352	1.79E-10	2.03E-08	yes	CABZ01088916.1
ENSDART00000175370	0.001	0.413333333	8.691161905	0.001058158	0.019542647	yes	FO834828.1
ENSDART00000175382	1.84	0.336666667	-2.450312974	0.015989548	0.163028125	no	CABZ01083102.1
ENSDART00000175391	0.61	0.143333333	-2.089435084	0.034185528	0.255675	no	CR846087.5
ENSDART00000175448	8.04	2.526666667	-1.669960154	0.000137417	0.003856929	yes	CABZ01068358.1

ENSDART00000175465	0.06	0.323333333	2.429987841	0.009675209	0.110359234	no	zgc:194629
ENSDART00000175535	0.426666667	1.256666667	1.558420713	0.004996261	0.069560803	no	CR388179.1
ENSDART00000175572	3.84	0.806666667	-2.251061764	0.000102177	0.002900437	yes	CABZ01059417.3
ENSDART00000175591	0.366666667	0.001	-8.518325308	0.000796711	0.015550785	yes	CABZ01087388.1
ENSDART00000175595	5.363333333	1.896666667	-1.499663768	0.016792118	0.165797275	no	CABZ01079873.1
ENSDART00000175603	0.11	0.37	1.750021747	0.000789296	0.015550785	yes	CR792418.4
ENSDART00000175628	7.796666667	0.8	-3.284785551	6.72E-07	3.81E-05	yes	BX000486.1
ENSDART00000175679	0.02	0.406666667	4.345774837	0.000673047	0.013780918	yes	CABZ01072845.1
ENSDART00000175691	0.096666667	0.693333333	2.842458723	4.80E-05	0.001640181	yes	CR384075.1
ENSDART00000175748	0.143333333	0.316666667	1.143590854	0.01119432	0.123731911	no	fancb
ENSDART00000175796	7.296666667	2.106666667	-1.792275491	8.72E-05	0.002622904	yes	CR762475.6
ENSDART00000175857	2.903333333	0.763333333	-1.927325121	0.018054999	0.171649203	no	BX323033.1
ENSDART00000175867	1.816666667	4.263333333	1.230688129	3.58E-10	3.89E-08	yes	CABZ01080224.1
ENSDART00000175900	0.433333333	0.001	-8.759333407	0.013115805	0.140614937	no	FO681360.2
ENSDART00000176026	0.15	0.001	-7.22881869	0.003333977	0.050474392	no	CABZ01044023.1
ENSDART00000176028	0.143333333	0.001	-7.163230349	0.044989232	0.306220987	no	BX927125.2
ENSDART00000176063	0.393333333	0.001	-8.619608644	0.047939537	0.320195091	no	BX901894.1
ENSDART00000176131	0.613333333	0.083333333	-2.879705766	0.021944403	0.18967861	no	CR735107.3
ENSDART00000176191	4.883333333	1.696666667	-1.525163103	0.013063665	0.140614937	no	CABZ01061478.1
ENSDART00000176229	1.14	0.001	-10.15481811	9.77E-07	5.30E-05	yes	FO818685.1
ENSDART00000176238	0.001	0.336666667	8.395177077	0.031293534	0.243602266	no	CABZ01056628.2
ENSDART00000176275	6.193333333	36.24333333	2.548927647	5.22E-19	4.35E-16	yes	BX000486.1
ENSDART00000176352	72.98333333	25.60333333	-1.511235395	0.002200948	0.036653114	yes	CT867953.1
ENSDART00000176355	1.966666667	5.143333333	1.386951202	6.87E-09	5.92E-07	yes	CABZ01039424.2
ENSDART00000176540	0.366666667	0.001	-8.518325308	0.00942484	0.109566911	no	CABZ01090818.1
ENSDART00000176616	5.683333333	0.786666667	-2.852912975	8.70E-05	0.002622904	yes	CR293509.4
ENSDART00000176635	5.153333333	1.77	-1.541756553	0.002500772	0.040564463	yes	CABZ01118317.1
ENSDART00000176661	0.406666667	0.001	-8.667702932	7.18E-09	5.98E-07	yes	FO704848.1
ENSDART00000176677	0.256666667	0.733333333	1.514573173	0.025432776	0.211066689	no	CU138506.3
ENSDART00000176682	0.116666667	0.001	-6.866248611	0.035018065	0.260856779	no	CABZ01059417.3
ENSDART00000176730	0.001	1.146666667	10.16323035	9.99E-11	1.25E-08	yes	CABZ010789

							89.1
ENSDART00000176871	18.04	4.32	-2.062096121	9.66E-08	6.35E-06	yes	CABZ01030033.1
ENSDART00000176956	0.183333333	0.001	-7.518325308	0.030995033	0.243598281	no	CABZ01056628.1
ENSDART00000177055	0.48	0.046666667	-3.362570079	0.000429579	0.010028866	yes	CABZ01063170.1
ENSDART00000177071	6.44	2.63	-1.291997889	0.020005149	0.179114201	no	CABZ01073546.1
ENSDART00000177136	0.001	0.113333333	6.824428435	0.00554817	0.075322436	no	CABZ01035191.4
ENSDART00000177239	2.253333333	0.293333333	-2.941447818	0.007041105	0.09187675	no	BX470140.1
ENSDART00000177366	3.873333333	0.973333333	-1.992569795	4.72E-05	0.001638686	yes	CR925768.4
ENSDART00000177369	2.183333333	0.076666667	-4.83178914	1.08E-07	6.91E-06	yes	CABZ01059417.3
ENSDART00000177544	15.63	1.736666667	-3.169925001	3.15E-07	1.83E-05	yes	CABZ01088053.1
ENSDART00000177561	0.15	0.326666667	1.122856748	0.018573562	0.174413873	no	BX640537.3
ENSDART00000177591	0.283333333	0.001	-8.14635653	0.035620482	0.262477771	no	CR790382.4
ENSDART00000177595	1.353333333	2.79	1.043747895	0.02385228	0.202663252	no	BX470214.1
ENSDART00000177599	1.25	0.226666667	-2.463283944	0.033105491	0.252724331	no	AL772298.4
ENSDART00000177676	1.776666667	0.413333333	-2.103795412	0.048319914	0.321875052	no	BX465848.6
ENSDART00000177724	0.176666667	0.47	1.411630898	0.002976095	0.046175686	yes	BX323984.1
ENSDART00000177745	1.16	0.42	-1.465663572	0.036433012	0.265334294	no	AL935044.5
ENSDART00000177749	5.816666667	1.49	-1.9648803	4.29E-06	0.00020618	yes	FO704848.1
ENSDART00000177793	0.023333333	0.28	3.584962501	0.008269854	0.10028202	no	CU855942.1
ENSDART00000177848	15.21333333	6.596666667	-1.205527179	0.017616393	0.170564923	no	CABZ01066434.1
ENSDART00000177891	1.036666667	0.306666667	-1.757208814	0.017928623	0.171592723	no	FO393393.1
ENSDART00000177901	0.001	0.176666667	7.464886049	0.007392695	0.09359646	no	CABZ01014197.1
ENSDART00000177950	0.96	0.001	-9.906890596	2.77E-15	1.15E-12	yes	BX640547.7
ENSDART00000178030	0.78	0.166666667	-2.22650853	0.040599476	0.288117875	no	CABZ01087388.1
ENSDART00000178064	1.603333333	3.363333333	1.068817375	0.000449082	0.010107892	yes	AL954146.3
ENSDART00000178146	0.026666667	0.77	4.851749041	0.002779251	0.043940317	yes	CABZ01069095.1
ENSDART00000178200	0.276666667	0.713333333	1.366427555	0.000372851	0.00887031	yes	FO704589.1
ENSDART00000178266	9.236666667	3.546666667	-1.38090856	0.035087221	0.260856779	no	FO704882.1
ENSDART00000178293	2.216666667	0.82	-1.434696025	0.043009115	0.296985487	no	BX664605.3
ENSDART00000178307	0.87	0.213333333	-2.027905997	0.013944158	0.145742702	no	CU467943.2
ENSDART00000178318	0.69	0.193333333	-1.835505962	0.008137141	0.099154045	no	CR751608.2
ENSDART00000178421	0.326666667	0.85	1.379643593	0.000915162	0.01731874	yes	BX936391.3

ENSDART00000178435	0.406666667	1.12	1.461580085	1.41E-07	8.61E-06	yes	CABZ01055408.1
ENSDART00000178438	0.523333333	0.02	-4.709658248	0.000786611	0.015550785	yes	BX957317.3
ENSDART00000178472	0.001	0.06	5.906890596	0.017865719	0.171592723	no	CU019646.1
ENSDART00000178534	0.876666667	0.226666667	-1.951456148	0.00994857	0.112450356	no	CABZ01067657.1
ENSDART00000178572	0.001	0.053333333	5.736965594	0.019762668	0.178220741	no	CU570781.2
ENSDART00000178577	1.22	4.823333333	1.983149368	3.98E-12	6.63E-10	yes	CABZ01072848.1
ENSDART00000178589	0.15	0.636666667	2.085575732	0.005667827	0.076119525	no	CU693368.2
ENSDART00000178656	15.41333333	33.15	1.104829973	5.26E-11	6.92E-09	yes	CABZ01080224.1
ENSDART00000178670	0.466666667	1.68	1.847996907	4.32E-11	5.99E-09	yes	BX936391.2
ENSDART00000178748	0.36	0.001	-8.491853096	0.013440489	0.142594987	no	CABZ01040021.1
ENSDART00000178805	1.146666667	0.116666667	-3.296981738	0.003017828	0.046534164	yes	BX936448.1
ENSDART00000178999	0.05	0.001	-5.64385619	0.024312623	0.204487985	no	CABZ01072077.1
ENSDART00000179136	0.001	0.25	7.965784285	0.027418587	0.220940742	no	CT573169.1
ENSDART00000179172	0.153333333	0.42	1.453717967	0.018712137	0.174413873	no	BX897682.2
ENSDART00000179265	5.463333333	1.83	-1.5779378	0.013345085	0.142461636	no	CABZ01063790.1
ENSDART00000179273	2.536666667	0.716666667	-1.823559794	0.009452875	0.109566911	no	BX649411.5
ENSDART00000179278	0.096666667	0.001	-6.594946589	0.019096817	0.176028966	no	CU570781.2
ENSDART00000179287	9.41	1.753333333	-2.424094424	2.97E-08	2.06E-06	yes	CR790382.5
ENSDART00000179301	0.001	0.266666667	8.058893689	0.003116893	0.047766861	yes	FP102311.1
ENSDART00000179304	0.203333333	0.001	-7.667702932	0.024817211	0.207335765	no	CR925768.4
ENSDART00000179343	0.856666667	0.093333333	-3.198269627	0.009746515	0.110667246	no	BX255935.4
ENSDART00000179424	9.953333333	3.276666667	-1.602950844	0.017744023	0.171137338	no	FO818732.1
ENSDART00000179466	0.383333333	0.913333333	1.252542032	0.032138001	0.24854714	no	BX005154.1
ENSDART00000179497	0.001	0.356666667	8.478432581	0.043037929	0.296985487	no	CR361542.3
ENSDART00000179576	2.4	0.76	-1.658963082	0.001063971	0.019542647	yes	BX296567.2
ENSDART00000179623	70.75666667	29.35333333	-1.269341741	0.00750824	0.094249162	no	CABZ01072255.2
ENSDART00000179638	1.973333333	0.46	-2.100928909	0.00089404	0.017060221	yes	CABZ01067170.1
TCONS_00000016	2.43	0.816666667	-1.573137065	0.004396878	0.063487867	no	-
TCONS_00001351	5.833333333	1.226666667	-2.249577251	0.000100695	0.00289123	yes	-
TCONS_00001517	2.356666667	0.336666667	-2.807354922	0.000738359	0.014995293	yes	-
TCONS_00005787	1.716666667	0.486666667	-1.818604063	0.016254754	0.163500813	no	-
TCONS_00006052	0.001	0.1	6.64385619	0.018698851	0.174413873	no	-
TCONS_00006313	3.736666667	1.416666667	-1.399251532	0.033906841	0.255079175	no	-
TCONS_00006524	1.276666667	0.396666667	-1.686382819	0.026788072	0.2172617	no	-

TCONS_00006542	0.64	1.476666667	1.206200388	9.26E-05	0.002727767	yes	-
TCONS_00008030	1.386666667	0.073333333	-4.2410081	0.008341812	0.100665928	no	-
TCONS_00008197	0.113333333	0.51	2.169925001	0.03363157	0.253811668	no	-
TCONS_00010602	0.633333333	0.18	-1.814968106	0.006762757	0.088912454	no	-
TCONS_00010870	3.64	7.37	1.017726169	1.22E-07	7.62E-06	yes	-
TCONS_00011728	0.84	0.053333333	-3.977279923	0.000165623	0.00449702	yes	-
TCONS_00014589	0.03	3.26	6.763765654	1.98E-44	4.95E-41	yes	-
TCONS_00014742	0.001	0.31	8.276124405	0.021294357	0.185611161	no	-
TCONS_00015168	0.416666667	0.02	-4.380821784	0.013675505	0.143535344	no	-
TCONS_00017420	3.393333333	1.086666667	-1.642793692	0.00225564	0.037315152	yes	-
TCONS_00017671	5.026666667	1.51	-1.735053473	0.003524927	0.052412302	no	-
TCONS_00018049	5.496666667	1.07	-2.360946196	0.016700233	0.165544368	no	-
TCONS_00018255	0.66	0.043333333	-3.928916902	0.011351648	0.124918133	no	-
TCONS_00018578	1.983333333	0.001	-10.95371145	1.73E-11	2.55E-09	yes	-
TCONS_00019790	1.336666667	0.353333333	-1.919537972	0.035387419	0.261821283	no	-
TCONS_00021840	0.513333333	0.026666667	-4.266786541	0.0173727	0.170184331	no	-
TCONS_00022620	0.386666667	0.033333333	-3.5360529	0.030063083	0.239164273	no	-
TCONS_00024839	3.336666667	1.03	-1.695763231	0.008511372	0.101244796	no	-
TCONS_00025683	4175.546667	1263.93	-1.724048524	1.01E-05	0.000436832	yes	-
TCONS_00025684	58.24333333	20.07	-1.537052308	0.008686903	0.102843056	no	-
TCONS_00026099	1.07	0.08	-3.741466986	0.004136869	0.060432158	no	-
TCONS_00027070	0.506666667	0.001	-8.984893108	0.00519067	0.071243374	no	-
TCONS_00027601	0.556666667	1.156666667	1.05508756	0.000585984	0.012618865	yes	-
TCONS_00027838	0.216666667	1.72	2.988859442	0.001355331	0.024182969	yes	-
TCONS_00029335	4.87	0.47	-3.37318911	3.62E-13	8.22E-11	yes	-
TCONS_00029336	2.706666667	0.383333333	-2.819845866	9.66E-09	7.54E-07	yes	-
TCONS_00029966	0.12	2.143333333	4.158749926	1.42E-15	7.09E-13	yes	-
TCONS_00030055	0.916666667	0.12	-2.933362807	0.026751351	0.2172617	no	-
TCONS_00030057	6.786666667	1.88	-1.851970494	5.44E-05	0.001765394	yes	-
TCONS_00030110	5.41	1.786666667	-1.598358094	0.009525048	0.109647782	no	-
TCONS_00036349	0.026666667	0.29	3.442943496	0.012962952	0.140179452	no	-
TCONS_00036351	0.21	0.67	1.673771768	0.003500593	0.05236217	no	-
TCONS_00037421	14.10666667	2.29	-2.622957623	1.66E-12	3.07E-10	yes	-
TCONS_00038949	0.756666667	0.001	-9.563514081	0.02514942	0.209410841	no	-
TCONS_00039155	13.82666667	3.356666667	-2.042352211	9.67E-05	0.002809922	yes	-
TCONS_00039837	0.41	3.616666667	3.140964822	4.93E-09	4.56E-07	yes	-
TCONS_00040852	0.03	0.4	3.736965594	0.022042815	0.189872252	no	-
TCONS_00041256	2.936666667	0.453333333	-2.695535368	0.000201461	0.005297356	yes	-
TCONS_00041817	0.833333333	0.296666667	-1.490050854	0.049500943	0.328651649	no	-
TCONS_00042166	0.053333333	0.376666667	2.820178962	0.010960144	0.122225183	no	-
TCONS_00042602	0.613333333	0.001	-9.26052755	0.004945414	0.069560803	no	-
TCONS_00044410	0.51	0.001	-8.994353437	0.024784056	0.207335765	no	-

TCONS_00044430	1.393333333	0.106666667	-3.707359132	0.013499548	0.142594987	no	-
TCONS_00044431	1.176666667	0.03	-5.293599372	0.000324288	0.007864775	yes	-
TCONS_00044494	4.95	1.14	-2.118394701	4.62E-05	0.00162541	yes	-
TCONS_00045363	4.91	2.163333333	-1.182467047	0.020615958	0.181333323	no	-
TCONS_00045712	0.07	0.596666667	3.091498354	0.002423931	0.039575032	yes	-
TCONS_00046249	4.573333333	1.846666667	-1.3083226	0.019740914	0.178220741	no	-
TCONS_00046349	0.46	0.013333333	-5.108524457	0.000477638	0.010653036	yes	-
TCONS_00046442	0.716666667	0.163333333	-2.133483005	0.039356283	0.281696259	no	-
TCONS_00046772	1.24	0.363333333	-1.770974486	0.00133635	0.024015838	yes	-
TCONS_00046866	0.446666667	0.001	-8.803054785	0.020529067	0.181207104	no	-
TCONS_00046887	1.173333333	0.493333333	-1.249978253	0.033183979	0.252724331	no	-
TCONS_00048649	0.29	0.023333333	-3.635588574	0.000933516	0.017533258	yes	-
TCONS_00049550	0.393333333	0.83	1.077358883	0.001280035	0.023170496	yes	-
TCONS_00050633	5.37	17.47	1.701885615	1.51E-20	1.89E-17	yes	-
TCONS_00050783	1.176666667	0.163333333	-2.848814529	0.007149388	0.092057583	no	-
TCONS_00051764	27.71666667	5.496666667	-2.334124865	0.001206742	0.022003225	yes	-
TCONS_00052313	4.476666667	0.001	-12.12820918	0.00014146	0.003926299	yes	-
TCONS_00052484	3.693333333	0.796666667	-2.212875358	0.000377872	0.008904943	yes	-
TCONS_00052585	0.486666667	0.001	-8.926790153	0.0006594	0.013726508	yes	-
TCONS_00053093	0.233333333	0.69	1.564203941	0.0205284	0.181207104	no	-
TCONS_00053305	0.606666667	0.096666667	-2.649813645	0.018071954	0.171649203	no	-
TCONS_00054110	0.5	1.653333333	1.72537762	0.003456173	0.05200915	no	-
TCONS_00054345	0.056666667	0.193333333	1.770518154	0.007625229	0.095239107	no	-
TCONS_00054435	1.193333333	0.3	-1.991962681	0.004563452	0.065514386	no	-
TCONS_00054852	0.56	0.033333333	-4.070389328	0.019091316	0.176028966	no	-
TCONS_00054942	0.006666667	0.533333333	6.321928095	1.08E-14	3.00E-12	yes	-
TCONS_00056892	4.196666667	10.36666667	1.304636297	3.98E-14	9.95E-12	yes	-
TCONS_00057087	4.743333333	0.996666667	-2.250718272	2.60E-08	1.85E-06	yes	-
TCONS_00057118	2.21	0.486666667	-2.183040501	0.00947416	0.109566911	no	-
TCONS_00057290	1.073333333	0.4	-1.424026283	0.02566498	0.211587856	no	-
TCONS_00057439	3.286666667	1.263333333	-1.379389798	0.047423799	0.31845336	no	-
TCONS_00058673	9.953333333	2.22	-2.164620083	2.41E-05	0.000940244	yes	-
TCONS_00058674	13.83	3.583333333	-1.948426997	0.000437402	0.010107892	yes	-
TCONS_00059403	0.99	0.001	-9.951284715	0.000183454	0.004927621	yes	-
TCONS_00059536	4.943333333	1.656666667	-1.577200841	0.0002352	0.006057018	yes	-
TCONS_00060060	1.6	3.35	1.06608919	1.78E-08	1.35E-06	yes	-
TCONS_00060162	0.68	0.04	-4.087462841	0.00255387	0.041158504	yes	-
TCONS_00060266	0.486666667	1.633333333	1.74681338	2.03E-08	1.49E-06	yes	-
TCONS_00061187	0.596666667	0.126666667	-2.235888264	0.03101051	0.243598281	no	-
TCONS_00062201	0.001	0.406666667	8.667702932	0.01461144	0.152080741	no	-
TCONS_00062691	0.466666667	0.001	-8.866248611	0.030560392	0.24158183	no	-
TCONS_00063727	1.323333333	0.286666667	-2.206730442	6.19E-05	0.001956227	yes	-

TCONS_00064933	0.41	0.001	-8.6794801	0.010492697	0.118066476	no	-
TCONS_00067059	0.001	0.476666667	8.896836931	0.005030885	0.069560803	no	-
TCONS_00067545	0.163333333	0.676666667	2.050626073	0.00026755	0.006750906	yes	-
TCONS_00067559	1.226666667	0.47	-1.384010604	0.042995162	0.296985487	no	-
TCONS_00069725	0.001	0.45	8.813781191	2.03E-05	0.000804886	yes	-
TCONS_00070223	1.766666667	0.35	-2.335603032	5.68E-05	0.001818991	yes	-
TCONS_00071196	0.556666667	0.12	-2.213779291	0.031385547	0.243602266	no	-
TCONS_00075414	0.406666667	0.046666667	-3.123382416	0.020326479	0.180695886	no	-

Table S2

GeneID	CK0dpa_fpkm	T7dpa_fpkm	log2(FC)	Pvalue	FDR	significant	Symbol
ENSDART00000007434	6.01	1.76	-1.771789562	1.25E-07	3.08E-05	yes	BX005421.1
ENSDART00000087449	0.096666667	0.696666667	2.849378137	4.37E-07	8.32E-05	yes	CR847968.1
ENSDART00000137680	0.023333333	0.41	4.135159583	0.000394765	0.02692079	yes	BX663503.1
ENSDART00000139663	0.443333333	1.67	1.913384358	0.012833577	0.305887694	no	CR391910.2
ENSDART00000140464	0.04	0.273333333	2.772589504	0.018646312	0.346523308	no	CR751602.1
ENSDART00000141092	5.733333333	1.773333333	-1.692910414	0.000102605	0.009329414	yes	BX469930.1
ENSDART00000144687	6.893333333	1.713333333	-2.008395921	3.52E-05	0.004318545	yes	CABZ01015815.1
ENSDART00000145771	3.116666667	1.25	-1.318075769	0.014901787	0.318120759	no	CT573344.1
ENSDART00000150088	0.07	1.226666667	4.131244533	0.000309718	0.02172448	yes	BX649485.2
ENSDART00000152652	0.363333333	0.026666667	-3.768184325	0.047962787	0.59770884	no	CT033796.2
ENSDART00000153176	1.573333333	0.593333333	-1.406909618	0.038770091	0.52585952	no	CR749163.1
ENSDART00000153298	10.13	34.13666667	1.752688017	4.82E-09	1.97E-06	yes	AL732488.1
ENSDART00000153409	1.203333333	0.276666667	-2.120815596	0.013027877	0.307533065	no	CR385078.4
ENSDART00000153498	0.25	0.903333333	1.853330351	0.01066065	0.272620037	no	BX294100.1
ENSDART00000153691	1.443333333	4.39	1.604816416	0.000118718	0.0097151	yes	CU928129.1
ENSDART00000153803	0.416666667	1.58	1.922958964	0.000131312	0.010399072	yes	CU466287.2
ENSDART00000154087	1.756666667	0.45	-1.964843555	0.020817718	0.363676093	no	AL772300.1
ENSDART00000154324	0.09	0.5	2.473931188	0.01415019	0.318120759	no	CR848791.3
ENSDART00000154808	0.39	0.001	-8.607330314	0.031336399	0.472005501	no	CR377211.3
ENSDART00000154910	3.796666667	1.35	-1.491773934	0.002986817	0.124281958	no	BX005421.1
ENSDART00000155403	0.026666667	0.346666667	3.700439718	0.023243449	0.388181407	no	AL954322.2
ENSDART00000155862	0.933333333	0.096666667	-3.271302022	0.027601528	0.440011378	no	CU683879.2
ENSDART00000156448	0.113333333	0.633333333	2.482392767	0.03582457	0.50018718	no	zgc:194207
ENSDART00000156594	0.64	0.036666667	-4.125530882	0.01377215	0.315987185	no	BX571794.3
ENSDART00000158007	13.52	4.403333333	-1.618427186	1.96E-05	0.002829021	yes	CU467961.1
ENSDART00000158696	1.16	0.001	-10.17990909	1.40E-07	3.12E-05	yes	mhc1zda
ENSDART00000161755	7.676666667	3.433333333	-1.160870074	0.028654425	0.450272629	no	CU681842.1
ENSDART00000163057	9.71	4.816666667	-1.011436209	0.005851191	0.19506191	no	FP101882.1
ENSDART00000163433	0.55	0.056666667	-3.278859373	0.040860059	0.545170891	no	FP016142.4
ENSDART00000164487	0.01	0.153333333	3.938599455	0.010229689	0.267168995	no	FP565451.1
ENSDART00000165941	0.52	0.001	-9.022367813	0.00011391	0.0097151	yes	CR382294.1
ENSDART00000167972	0.583333333	1.273333333	1.126217716	0.009707391	0.264796056	no	CU929418.2
ENSDART00000169453	4.34	12.56333333	1.533452346	7.15E-08	1.95E-05	yes	CU469577.1
ENSDART00000170230	0.036666667	0.46	3.649092838	0.025304712	0.4095959	no	BX682557.1
ENSDART00000172918	0.246666667	1.306666667	2.405256478	0.003117934	0.127575454	no	BX005228.1
ENSDART00000173489	2.43	0.77	-1.658025963	0.019629607	0.354343274	no	BX957297.2
ENSDART00000173958	0.61	0.001	-9.252665432	0.003937063	0.148699836	no	CR318601.1
ENSDART00000174069	12.06666667	3.173333333	-1.926956219	4.03E-05	0.004712892	yes	CABZ01015815.1
ENSDART00000174479	0.11	0.45	2.032421478	0.002008167	0.093019808	no	CABZ01077124.2

ENSDART00000174625	0.16	0.001	-7.321928095	0.035858633	0.50018718	no	CABZ01113900.1
ENSDART00000174821	1.02	0.386666667	-1.399406848	0.014862678	0.318120759	no	CR792455.2
ENSDART00000174877	0.343333333	0.001	-8.423466121	0.01511416	0.319872963	no	CT009596.2
ENSDART00000175018	0.233333333	0.59	1.338322533	0.016744422	0.324313784	no	CABZ01007102.1
ENSDART00000175043	0.233333333	1.036666667	2.151487753	0.044369221	0.566814611	no	CABZ01040021.1
ENSDART00000175077	0.001	1.196666667	10.22480563	5.72E-10	2.81E-07	yes	CABZ01079894.1
ENSDART00000175116	0.001	0.16	7.321928095	0.016559362	0.324313784	no	CABZ01100207.1
ENSDART00000175180	0.773333333	0.26	-1.572578776	0.049706581	0.605975529	no	BX000981.13
ENSDART00000175275	0.001	0.36	8.491853096	0.014551823	0.318120759	no	AL928838.1
ENSDART00000175391	0.61	0.19	-1.682809824	0.049860308	0.605975529	no	CR846087.5
ENSDART00000175465	0.06	0.4	2.736965594	0.022473007	0.380491259	no	zgc:194629
ENSDART00000175535	0.426666667	1.29	1.596189756	0.019290176	0.350795424	no	CR388179.1
ENSDART00000175572	3.84	1.6	-1.263034406	0.006625517	0.209947861	no	CABZ01059417.3
ENSDART00000175591	0.366666667	0.053333333	-2.781359714	0.042025884	0.551268699	no	CABZ01087388.1
ENSDART00000175595	5.363333333	2.516666667	-1.091615777	0.016472039	0.324313784	no	CABZ01079873.1
ENSDART00000175628	7.796666667	1.97	-1.984661826	8.31E-05	0.008341625	yes	BX000486.1
ENSDART00000175689	0.15	0.001	-7.22881869	0.04859064	0.602474853	no	CABZ01113900.1
ENSDART00000175691	0.096666667	0.5	2.370837695	0.007014139	0.215246392	no	CR384075.1
ENSDART00000175796	7.296666667	2.816666667	-1.373248708	0.00011763	0.0097151	yes	CR762475.6
ENSDART00000175857	2.903333333	0.923333333	-1.652786743	0.008622295	0.249032181	no	BX323033.1
ENSDART00000176028	0.143333333	0.001	-7.163230349	0.047905358	0.59770884	no	BX927125.2
ENSDART00000176275	6.193333333	21.88333333	1.821044509	1.73E-08	5.32E-06	yes	BX000486.1
ENSDART00000176326	0.056666667	0.146666667	1.371968777	0.031338858	0.472005501	no	AL935115.2
ENSDART00000176355	1.966666667	4.663333333	1.245609103	1.26E-05	0.001927879	yes	CABZ01039424.2
ENSDART00000176616	5.683333333	1.853333333	-1.616614951	0.004733834	0.16842845	no	CR293509.4
ENSDART00000176635	5.153333333	2.253333333	-1.193445168	0.001427821	0.071554742	no	CABZ01118317.1
ENSDART00000176651	0.316666667	0.75	1.243925583	0.035702113	0.50018718	no	CABZ01040256.2
ENSDART00000176730	0.001	0.65	9.344295908	2.19E-05	0.002989395	yes	CABZ01078989.1
ENSDART00000176956	0.183333333	0.001	-7.518325308	0.028321459	0.44857537	no	CABZ01056628.1
ENSDART00000177055	0.48	0.1	-2.263034406	0.014849459	0.318120759	no	CABZ01063170.1
ENSDART00000177136	0.001	0.126666667	6.984893108	0.00421511	0.154449174	no	CABZ01035191.4
ENSDART00000177154	0.001	0.486666667	8.926790153	0.018772953	0.346523308	no	BX324006.5
ENSDART00000177211	0.116666667	0.001	-6.866248611	0.020140712	0.355722646	no	FO704769.1
ENSDART00000177676	1.776666667	0.52	-1.772589504	0.025359909	0.4095959	no	BX465848.6
ENSDART00000177749	5.816666667	2.876666667	-1.015794572	0.006787403	0.210925006	no	FO704848.1
ENSDART00000177950	0.96	0.001	-9.906890596	4.31E-17	5.29E-14	yes	BX640547.7
ENSDART00000178030	0.78	0.113333333	-2.782901878	0.003446174	0.134029289	no	CABZ01087388.1
ENSDART00000178105	0.973333333	0.116666667	-3.060541542	0.019168211	0.350795424	no	CABZ01078614.2
ENSDART00000178200	0.276666667	0.826666667	1.579156879	0.001387597	0.071554742	no	FO704589.1
ENSDART00000178293	2.216666667	0.983333333	-1.172639386	0.028795439	0.450272629	no	BX664605.3
ENSDART00000178307	0.87	0.363333333	-1.259721672	0.048917691	0.603482065	no	CU467943.2
ENSDART00000178421	0.326666667	0.823333333	1.333657387	0.006153946	0.198788641	no	BX936391.3

ENSDART00000178490	0.303333333	0.001	-8.244760234	0.011625633	0.285721254	no	CR456635.1
ENSDART00000178534	0.876666667	0.24	-1.868993988	0.031772581	0.473257685	no	CABZ01067657.1
ENSDART00000178577	1.22	2.446666667	1.003936415	0.000649759	0.038906311	yes	CABZ01072848.1
ENSDART00000178589	0.15	0.89	2.568842835	0.00142818	0.071554742	no	CU693368.2
ENSDART00000178636	0.12	0.001	-6.906890596	0.032509669	0.48079058	no	AL954138.2
ENSDART00000178670	0.466666667	1.55	1.731803889	5.86E-09	2.06E-06	yes	BX936391.2
ENSDART00000178748	0.36	0.001	-8.491853096	0.011554288	0.285721254	no	CABZ01040021.1
ENSDART00000178865	3.243333333	0.81	-2.001483491	0.002578399	0.11303518	no	FO681289.2
ENSDART00000178999	0.05	0.001	-5.64385619	0.022358898	0.380491259	no	CABZ01072077.1
ENSDART00000179105	0.19	0.71	1.901819606	0.040193976	0.539214274	no	CR853282.1
ENSDART00000179116	1.686666667	0.556666667	-1.599289282	0.003280584	0.12990053	no	AL954146.2
ENSDART00000179265	5.463333333	2.433333333	-1.166847485	0.013623385	0.315522727	no	CABZ01063790.1
ENSDART00000179278	0.096666667	0.001	-6.594946589	0.016874505	0.324313784	no	CU570781.2
ENSDART00000179438	1.093333333	0.323333333	-1.757639162	0.017220759	0.327728398	no	BX465848.5
ENSDART00000179444	0.14	0.32	1.192645078	0.014058874	0.318120759	no	CABZ01053592.1
ENSDART00000179453	2.906666667	1.063333333	-1.450771711	0.036859895	0.508376641	no	CT573356.1
ENSDART00000179474	0.001	0.263333333	8.040746342	0.008353778	0.246210381	no	BX936391.3
ENSDART00000179504	0.14	0.001	-7.129283017	0.001289779	0.068834966	no	CABZ01072077.1
ENSDART00000179576	2.4	1.123333333	-1.095248315	0.006063973	0.198494051	no	BX296567.2
TCONS_00000016	2.43	1.063333333	-1.192362391	0.005879667	0.19506191	no	-
TCONS_00001517	2.356666667	0.736666667	-1.677663845	0.016726139	0.324313784	no	-
TCONS_00005787	1.716666667	0.373333333	-2.2010737	0.003494043	0.134029289	no	-
TCONS_00006042	0.19	1.113333333	2.550814278	0.009562874	0.263784885	no	-
TCONS_00006389	0.001	0.63	9.299208018	0.031185731	0.472005501	no	-
TCONS_00007545	0.8	0.173333333	-2.206450877	0.015547823	0.323473784	no	-
TCONS_00007557	0.856666667	1.793333333	1.065837813	0.013235459	0.309457643	no	-
TCONS_00008660	0.803333333	0.273333333	-1.555337332	0.016902413	0.324313784	no	-
TCONS_00010602	0.633333333	0.213333333	-1.569855608	0.003201816	0.128859976	no	-
TCONS_00014589	0.03	0.69	4.523561956	6.44E-20	1.58E-16	yes	-
TCONS_00017420	3.393333333	1.58	-1.102778597	0.010467223	0.270495091	no	-
TCONS_00018049	5.496666667	1.766666667	-1.637527134	0.017457977	0.329687183	no	-
TCONS_00018159	2.476666667	1.18	-1.06961285	0.033007023	0.485223005	no	-
TCONS_00018577	0.73	0.001	-9.511752654	0.002697326	0.116174316	no	-
TCONS_00020260	1.346666667	0.263333333	-2.354430735	0.009233232	0.260546952	no	-
TCONS_00027070	0.506666667	0.053333333	-3.247927513	0.042171379	0.551268699	no	-
TCONS_00027601	0.556666667	1.36	1.288721049	0.00058158	0.035694448	yes	-
TCONS_00029335	4.87	1.796666667	-1.438599	0.000239532	0.017295598	yes	-
TCONS_00029336	2.706666667	1.286666667	-1.07287888	0.03328993	0.486468919	no	-
TCONS_00029966	0.12	1.7	3.824428435	2.79E-12	1.71E-09	yes	-
TCONS_00036349	0.026666667	0.356666667	3.741466986	0.019908326	0.355722646	no	-
TCONS_00036351	0.21	0.82	1.965234582	0.004587946	0.165638329	no	-
TCONS_00037387	0.67	0.176666667	-1.923131237	0.016111482	0.324313784	no	-

TCONS_00037421	14.10666667	6.15	-1.197718811	0.000704183	0.040369176	yes	-
TCONS_00038949	0.756666667	0.001	-9.563514081	0.021985484	0.380101149	no	-
TCONS_00044430	1.393333333	0.216666667	-2.684991319	0.030354175	0.466345645	no	-
TCONS_00044431	1.176666667	0.156666667	-2.908935522	0.020887303	0.363676093	no	-
TCONS_00044494	4.95	1.84	-1.427722759	0.000577282	0.035694448	yes	-
TCONS_00045363	4.91	2.366666667	-1.052866501	0.001795929	0.086451104	no	-
TCONS_00045778	0.243333333	0.613333333	1.333737397	0.042588828	0.553204085	no	-
TCONS_00046349	0.46	0.113333333	-2.021061616	0.038679368	0.52585952	no	-
TCONS_00048399	0.646666667	0.23	-1.491388385	0.016427089	0.324313784	no	-
TCONS_00048649	0.29	0.03	-3.273018494	1.13E-06	0.000184127	yes	-
TCONS_00049225	1.63	0.376666667	-2.113511693	0.036856283	0.508376641	no	-
TCONS_00050633	5.37	19.37	1.850829961	2.36E-12	1.71E-09	yes	-
TCONS_00050783	1.176666667	0.236666667	-2.313777254	0.016909232	0.324313784	no	-
TCONS_00051226	0.433333333	0.01	-5.437405312	0.000536909	0.034687134	yes	-
TCONS_00051830	0.593333333	0.07	-3.083416008	0.00908879	0.259453257	no	-
TCONS_00052484	3.693333333	1.546666667	-1.255761171	0.004959168	0.171475452	no	-
TCONS_00054345	0.056666667	0.353333333	2.640457613	0.000707077	0.040369176	yes	-
TCONS_00054435	1.193333333	0.42	-1.506535854	0.008424306	0.246210381	no	-
TCONS_00054942	0.006666667	0.316666667	5.569855608	5.35E-07	9.39E-05	yes	-
TCONS_00056520	0.001	0.22	7.781359714	0.005312136	0.181129076	no	-
TCONS_00057087	4.743333333	2.106666667	-1.170939198	0.000790794	0.043142199	yes	-
TCONS_00057233	0.64	0.001	-9.321928095	0.004855103	0.170275406	no	-
TCONS_00059403	0.99	0.176666667	-2.486398666	0.027348046	0.43881995	no	-
TCONS_00059967	2.116666667	6.533333333	1.626025157	4.41E-07	8.32E-05	yes	-
TCONS_00059970	1.45	4.08	1.492516252	0.000170093	0.012653924	yes	-
TCONS_00060162	0.68	0.05	-3.765534746	0.00221934	0.100897753	no	-
TCONS_00062691	0.466666667	0.001	-8.866248611	0.025299112	0.4095959	no	-
TCONS_00063057	1.04	2.323333333	1.159612627	8.49E-05	0.008341625	yes	-
TCONS_00063634	0.793333333	0.096666667	-3.036836768	0.01796159	0.336608423	no	-
TCONS_00063727	1.323333333	0.44	-1.588601078	0.000439334	0.029150402	yes	-
TCONS_00064933	0.41	2.33	2.50663414	0.009925888	0.267168995	no	-
TCONS_00067059	0.001	0.416666667	8.702749879	0.015330681	0.321682234	no	-
TCONS_00067060	0.87	0.143333333	-2.601641242	0.042215281	0.551268699	no	-
TCONS_00069725	0.001	0.686666667	9.423466121	3.49E-05	0.004318545	yes	-
TCONS_00070224	0.38	0.001	-8.569855608	5.30E-05	0.005918683	yes	-
TCONS_00071549	1.916666667	0.66	-1.538061526	0.02294893	0.385887828	no	-
TCONS_00076154	0.156666667	0.001	-7.291554446	0.008417328	0.246210381	no	-

Table S3

GeneID	T3dpa_fpkm	T7dpa_fpkm	log2(FC)	Pvalue	FDR	significant	Symbol
ENSDART00000007434	0.813333333	1.76	1.113656782	0.010685968	0.34967118	no	BX005421.1
ENSDART00000124127	0.31	0.001	-8.276124405	0.030627567	0.536533287	no	CU179643.1
ENSDART00000142301	0.58	0.001	-9.17990909	0.025421939	0.494771036	no	BX005355.1
ENSDART00000144731	0.001	0.223333333	7.803054785	0.049851153	0.717265134	no	CR377211.3
ENSDART00000145442	0.56	0.116666667	-2.263034406	0.041431751	0.672575419	no	CR936321.1
ENSDART00000145874	6.726666667	16.46666667	1.291584867	0.00166697	0.093166873	no	BX936337.1
ENSDART00000148196	5.576666667	18.89666667	1.760656824	2.36E-06	0.000796007	yes	AL929237.1
ENSDART00000148329	0.306666667	0.83	1.436439976	0.02081609	0.470239717	no	BX088709.1
ENSDART00000152541	0.976666667	2.003333333	1.036464326	0.049724919	0.717265134	no	BX548015.2
ENSDART00000153479	0.01	0.186666667	4.222392421	0.012090161	0.367994263	no	CR377211.3
ENSDART00000154324	1.006666667	0.5	-1.009586049	0.024126967	0.486509179	no	CR848791.3
ENSDART00000155205	2.983333333	0.153333333	-4.282181916	2.62E-06	0.000796007	yes	CU927934.1
ENSDART00000155541	5.64	37.85	2.746526232	3.28E-09	1.99E-06	yes	CU693494.2
ENSDART00000155813	0.283333333	0.001	-8.14635653	0.001480324	0.092425355	no	CU927934.3
ENSDART00000155964	0.186666667	0.013333333	-3.807354922	0.018891503	0.450988331	no	CR628341.1
ENSDART00000158002	0.206666667	0.02	-3.36923381	0.008715565	0.298907058	no	CR812832.1
ENSDART00000158007	2.066666667	4.403333333	1.091290346	0.048276556	0.717265134	no	CU467961.1
ENSDART00000160098	0.076666667	0.903333333	3.558587085	0.024328268	0.486509179	no	BX901923.2
ENSDART00000165941	0.22	0.001	-7.781359714	0.017601629	0.432928963	no	CR382294.1
ENSDART00000169452	0.013333333	0.286666667	4.426264755	0.021159784	0.470239717	no	CR848841.2
ENSDART00000172786	0.62	0.203333333	-1.608421474	0.049227905	0.717265134	no	CT574585.2
ENSDART00000174069	1.13	3.173333333	1.4896763	0.008588795	0.298907058	no	CABZ01015815.1
ENSDART00000174163	1.373333333	0.326666667	-2.071790683	0.017016545	0.428631707	no	CABZ01068273.1
ENSDART00000174415	4.23	8.59	1.022000468	0.044349387	0.694197185	no	CR376839.1
ENSDART00000174436	0.016666667	0.716666667	5.426264755	0.020250621	0.469024085	no	FP101914.2
ENSDART00000174739	0.106666667	0.496666667	2.21916852	0.035603812	0.606260717	no	CABZ01002339.2
ENSDART00000174873	0.19	0.52	1.452512205	0.047469026	0.717265134	no	CABZ01004903.1
ENSDART00000174983	1.163333333	2.913333333	1.324406243	0.017426259	0.432928963	no	CABZ01077121.1
ENSDART00000175043	0.016666667	1.036666667	5.958842675	0.000672028	0.046753923	yes	CABZ01040021.1
ENSDART00000175059	0.62	2.473333333	1.996116566	2.38E-07	9.66E-05	yes	BX571711.3
ENSDART00000175370	0.413333333	0.001	-8.691161905	0.001781799	0.096008906	no	FO834828.1
ENSDART00000175489	0.093333333	0.001	-6.544320516	0.045181412	0.697451743	no	BX546499.3
ENSDART00000175491	1.813333333	0.816666667	-1.150824902	0.000667696	0.046753923	yes	BX936350.2

ENSDART00000175603	0.37	0.136666667	-1.436863862	0.011260089	0.351516867	no	CR792418.4
ENSDART00000175679	0.406666667	0.02	-4.345774837	0.001029046	0.067722344	no	CABZ01072845.1
ENSDART00000175748	0.316666667	0.126666667	-1.321928095	0.014822422	0.416437168	no	fancb
ENSDART00000175900	0.001	0.4	8.64385619	0.012268651	0.368816866	no	FO681360.2
ENSDART00000176026	0.001	0.106666667	6.736965594	0.000555998	0.04102589	yes	CABZ01044023.1
ENSDART00000176069	0.743333333	0.09	-3.046012398	0.009871552	0.329277099	no	CABZ01057364.1
ENSDART00000176229	0.001	0.74	9.531381461	1.16E-14	9.43E-12	yes	FO818685.1
ENSDART00000176548	2.23	1.096666667	-1.023918627	0.005356636	0.228831751	no	CABZ01066326.1
ENSDART00000176651	0.14	0.75	2.421463768	0.022966791	0.485277377	no	CABZ01040256.2
ENSDART00000176661	0.001	0.153333333	7.26052755	0.023621789	0.486509179	no	FO704848.1
ENSDART00000176871	4.32	11.42666667	1.403301391	0.000444838	0.033849364	yes	CABZ01030033.1
ENSDART00000177123	0.66	1.836666667	1.476551888	0.021629096	0.470239717	no	CABZ01101991.1
ENSDART00000177154	0.001	0.486666667	8.926790153	0.027475336	0.504756037	no	BX324006.5
ENSDART00000177161	0.001	0.35	8.451211112	0.01368059	0.39657425	no	CABZ01084143.1
ENSDART00000177366	0.973333333	2.94	1.594810287	0.000335776	0.030282043	yes	CR925768.4
ENSDART00000177369	0.076666667	1.36	4.148863386	8.36E-05	0.011314184	yes	CABZ01059417.3
ENSDART00000177544	1.736666667	12.07666667	2.797829528	1.74E-07	8.48E-05	yes	CABZ01088053.1
ENSDART00000177561	0.326666667	0.043333333	-2.914270126	0.002312397	0.117305977	no	BX640537.3
ENSDART00000177793	0.28	0.026666667	-3.392317423	0.026211689	0.494771036	no	CU855942.1
ENSDART00000178105	0.57	0.116666667	-2.288569498	0.032332935	0.559908895	no	CABZ01078614.2
ENSDART00000178146	0.77	0.001	-9.588714636	0.004440911	0.196611242	no	CABZ01069095.1
ENSDART00000178572	0.053333333	0.001	-5.736965594	0.027777129	0.504756037	no	CU570781.2
ENSDART00000178766	0.263333333	0.116666667	-1.174497731	0.049851175	0.717265134	no	FO904911.1
ENSDART00000179287	1.753333333	6.113333333	1.801858934	1.06E-05	0.002586177	yes	CR790382.5
ENSDART00000179304	0.001	0.156666667	7.291554446	0.042889144	0.691622945	no	CR925768.4
ENSDART00000179343	0.093333333	0.7	2.906890596	0.015203182	0.416437168	no	BX255935.4
ENSDART00000179468	0.466666667	1.34	1.521768674	0.002857227	0.141986698	no	BX640547.7
ENSDART00000179504	0.07	0.001	-6.129283017	0.004113551	0.193226855	no	CABZ01072077.1
TCONS_00001351	1.226666667	5.336666667	2.121195636	2.88E-05	0.005396138	yes	-
TCONS_00003006	0.066666667	0.366666667	2.459431619	0.032421829	0.559908895	no	-

TCONS_00006042	0.136666667	1.113333333	3.026152288	0.01120594	0.351516867	no	-
TCONS_00006052	0.1	0.001	-6.64385619	0.024311173	0.486509179	no	-
TCONS_00014393	1.373333333	2.933333333	1.094859186	0.02838438	0.505864654	no	-
TCONS_00014589	3.26	0.69	-2.240203697	6.18E-15	7.53E-12	yes	-
TCONS_00014742	0.31	0.001	-8.276124405	0.02616544	0.494771036	no	-
TCONS_00015085	0.63	0.046666667	-3.754887502	0.024927574	0.489505188	no	-
TCONS_00017671	1.51	4.573333333	1.598697526	0.001622285	0.093166873	no	-
TCONS_00018577	0.546666667	0.001	-9.094517599	4.43E-05	0.007185106	yes	-
TCONS_00018578	0.001	0.69	9.430452552	0.001964332	0.101769115	no	-
TCONS_00020260	0.653333333	0.263333333	-1.310929096	0.040523904	0.666727746	no	-
TCONS_00025684	20.07	42.45333333	1.080837218	0.016507385	0.427611518	no	-
TCONS_00025686	23.06333333	51.62	1.162329101	0.022703433	0.484937362	no	-
TCONS_00025687	40.69333333	81.79	1.007132002	0.03620512	0.612218525	no	-
TCONS_00029129	2.083333333	4.726666667	1.181929438	0.01622225	0.427611518	no	-
TCONS_00029335	0.47	1.796666667	1.93459011	2.49E-05	0.005061016	yes	-
TCONS_00029336	0.383333333	1.286666667	1.746966986	0.003701292	0.180252931	no	-
TCONS_00030057	1.88	4.656666667	1.308564953	0.001813721	0.096008906	no	-
TCONS_00030110	1.786666667	4.6	1.364363361	0.005720966	0.236111071	no	-
TCONS_00035535	0.513333333	0.176666667	-1.538866086	0.000373039	0.031322406	yes	-
TCONS_00037421	2.29	6.15	1.425238812	3.33E-05	0.005792808	yes	-
TCONS_00039837	3.616666667	1.066666667	-1.761551232	5.02E-05	0.007579358	yes	-
TCONS_00041256	0.453333333	1.52	1.745427173	0.016376249	0.427611518	no	-
TCONS_00045712	0.596666667	0.19	-1.650925763	0.045542023	0.697451743	no	-
TCONS_00046249	1.846666667	3.793333333	1.038542676	0.018684605	0.450465471	no	-
TCONS_00047310	0.206666667	0.63	1.608046114	0.043571104	0.693435551	no	-
TCONS_00047900	0.563333333	0.183333333	-1.619519723	0.037210398	0.624878061	no	-
TCONS_00047994	0.32	0.883333333	1.464886049	0.026562925	0.496259093	no	-
TCONS_00049212	0.403333333	1.016666667	1.333802195	0.024425366	0.486509179	no	-
TCONS_00051226	0.163333333	0.01	-4.029747343	0.021587284	0.470239717	no	-
TCONS_00051764	5.496666667	21.90666667	1.994741082	0.001683508	0.093166873	no	-
TCONS_00052313	0.001	2.866666667	11.48515844	0.000159057	0.016845951	yes	-
TCONS_00057087	0.996666667	2.106666667	1.079779074	0.01502522	0.416437168	no	-
TCONS_00057233	0.263333333	0.001	-8.040746342	0.028091666	0.505864654	no	-
TCONS_00059536	1.656666667	3.5	1.079071571	0.007327869	0.270353958	no	-
TCONS_00059967	2.153333333	6.533333333	1.601247584	0.000144944	0.016806592	yes	-
TCONS_00059970	1.7	4.08	1.263034406	0.020417476	0.469024085	no	-
TCONS_00060266	1.633333333	0.693333333	-1.236198221	1.12E-05	0.002586177	yes	-
TCONS_00060703	0.243333333	1.226666667	2.333737397	0.02269883	0.484937362	no	-
TCONS_00063057	1.063333333	2.323333333	1.127602232	0.025692323	0.494771036	no	-
TCONS_00064933	0.001	2.33	11.18611424	3.49E-20	8.51E-17	yes	-
TCONS_00070223	0.35	1.116666667	1.673771768	0.006851864	0.260692016	no	-
TCONS_00075414	0.046666667	0.55	3.558967292	0.000728885	0.049300987	yes	-

Table S4

	GO ID	Description	cis-mRNA (448)	pvalue	p.adjust
the target genes of DE lncRNAs（cis） GO Enrichment (Biological Process)					
1	GO:0043234	protein complex	70	0.000768679	0.049194378
2	GO:0031227	intrinsic component of endoplasmic reticulum membrane	6	0.000872798	0.049194378
3	GO:0005793	endoplasmic reticulum-Golgi intermediate compartment	4	0.00094044	0.049194378
4	GO:0032991	macromolecular complex	97	0.000978993	0.049194378
5	GO:0000123	histone acetyltransferase complex	5	0.002083464	0.083755258
6	GO:0008023	transcription elongation factor complex	4	0.004246158	0.142246291
7	GO:0005789	endoplasmic reticulum membrane	7	0.006257513	0.179680025
8	GO:0005657	replication fork	2	0.008975421	0.20260756
9	GO:0031248	protein acetyltransferase complex	5	0.011155905	0.20260756
10	GO:1902493	acetyltransferase complex	5	0.011155905	0.20260756
11	GO:0015934	large ribosomal subunit	3	0.012200253	0.20260756
12	GO:0000777	condensed chromosome kinetochore	2	0.0131888	0.20260756
13	GO:0000779	condensed chromosome, centromeric region	2	0.0131888	0.20260756
14	GO:0034704	calcium channel complex	5	0.014617644	0.20260756
15	GO:0042175	nuclear outer membrane-endoplasmic reticulum membrane network	16	0.01514811	0.20260756
16	GO:0034703	cation channel complex	6	0.016127965	0.20260756
17	GO:0070461	SAGA-type complex	2	0.018089069	0.21387664
18	GO:0044432	endoplasmic reticulum part	7	0.019162506	0.213981315
19	GO:0044427	chromosomal part	16	0.021489059	0.223005497
20	GO:0000776	kinetochore	4	0.022189602	0.223005497
21	GO:0031091	platelet alpha granule	1	0.03093709	0.241522632
22	GO:0042611	MHC protein complex	4	0.031672309	0.241522632
23	GO:0030424	axon	3	0.032909093	0.241522632
24	GO:0033267	axon part	3	0.032909093	0.241522632
25	GO:0034702	ion channel complex	6	0.0335913	0.241522632
26	GO:1902495	transmembrane transporter complex	6	0.035443027	0.241522632
27	GO:1990351	transporter complex	6	0.035443027	0.241522632
28	GO:0000808	origin recognition complex	2	0.036458711	0.241522632
29	GO:0005783	endoplasmic reticulum	7	0.037087462	0.241522632
30	GO:0098796	membrane protein complex	14	0.037500351	0.241522632
31	GO:0005654	nucleoplasm	12	0.040013414	0.241522632
32	GO:0044451	nucleoplasm part	12	0.040013414	0.241522632
33	GO:0031300	intrinsic component of organelle membrane	8	0.040283474	0.241522632
34	GO:0044437	vacuolar part	3	0.040854575	0.241522632
35	GO:0036338	viral membrane	2	0.043664669	0.250759957

Table S5

the target genes of DE lncRNAs（cis） Pathway Enrichment

	Pathway	cis-mRNA (367)	All (14723)	Pvalue	Qvalue	Pathway ID
1	Proteasome	9	91	0.000436453	0.03133835	ko03050
2	Gap junction	19	323	0.000493517	0.03133835	ko04540
3	Wnt signaling pathway	25	504	0.000874514	0.03702107	ko04310
4	Toll-like receptor signaling pathway	15	261	0.002360868	0.07495756	ko04620
5	N-Glycan biosynthesis	9	132	0.005814242	0.14768175	ko00510
6	Peroxisome	11	194	0.009498752	0.20105692	ko04146
7	Jak-STAT signaling pathway	18	412	0.01542273	0.22160747	ko04630
8	Cell cycle	16	361	0.01920199	0.22160747	ko04110
9	Cell adhesion molecules (CAMs)	22	551	0.02045628	0.22160747	ko04514
10	Oocyte meiosis	15	335	0.02114916	0.22160747	ko04114
11	Nitrogen metabolism	4	43	0.02182489	0.22160747	ko00910
12	MAPK signaling pathway	34	955	0.02282244	0.22160747	ko04010
13	Biosynthesis of amino acids	10	193	0.02304499	0.22160747	ko01230
14	Caffeine metabolism	2	10	0.02442917	0.22160747	ko00232
15	RIG-I-like receptor signaling pathway	9	172	0.0285858	0.24202644	ko04622
16	Ras signaling pathway	2	12	0.03467532	0.25904504	ko04014
17	Circadian entrainment	2	12	0.03467532	0.25904504	ko04713
18	Basal transcription factors	6	100	0.03889271	0.27440968	ko03022
19	Hedgehog signaling pathway	7	130	0.04418766	0.29535962	ko04340
20	VEGF signaling pathway	10	220	0.04935045	0.30327265	ko04370

Table S6

LncRNA	chr	start	end	strand	GeneID	chr	start	end	strand	up/down_Stream	distance	correlation
ENSDART00000154324	15	17440345	17442041	+	ENSDART00000018461	15	17407297	17437330	+	DOWNSTREAM	3014	0.858410361516328
